# A genome-wide association meta-analysis on lipoprotein (a) concentrations adjusted for apolipoprotein (a) isoforms[S]

**DOI:** 10.1194/jlr.M076232

**Published:** 2017-05-16

**Authors:** Salome Mack, Stefan Coassin, Rico Rueedi, Noha A. Yousri, Ilkka Seppälä, Christian Gieger, Sebastian Schönherr, Lukas Forer, Gertraud Erhart, Pedro Marques-Vidal, Janina S. Ried, Gerard Waeber, Sven Bergmann, Doreen Dähnhardt, Andrea Stöckl, Olli T. Raitakari, Mika Kähönen, Annette Peters, Thomas Meitinger, Konstantin Strauch, KORA-Study Group, Ludmilla Kedenko, Bernhard Paulweber, Terho Lehtimäki, Steven C. Hunt, Peter Vollenweider, Claudia Lamina, Florian Kronenberg

**Affiliations:** Division of Genetic Epidemiology,* Department of Medical Genetics, Molecular and Clinical Pharmacology, Medical University of Innsbruck, 6020 Innsbruck, Austria; Department of Computational Biology,† University of Lausanne, 1015 Lausanne, Switzerland; Swiss Institute of Bioinformatics,§ 1015 Lausanne, Switzerland; Department of Physiology and Biophysics,‡ Weill Cornell Medical College-Qatar, Doha, Qatar; Department of Computer and Systems Engineering,|| Alexandria University, 21526 Alexandria, Egypt; Department of Clinical Chemistry,# Fimlab Laboratories and University of Tampere School of Medicine, 33520 Tampere, Finland; Institute of Genetic Epidemiology,$ Helmholtz Zentrum München-German Research Center for Environmental Health, 85764 Neuherberg, Germany; Institute of Epidemiology II,** Helmholtz Zentrum München-German Research Center for Environmental Health, 85764 Neuherberg, Germany; Research Unit of Molecular Epidemiology,†† Helmholtz Zentrum München-German Research Center for Environmental Health, 85764 Neuherberg, Germany; Institute of Human Genetics, §§§ Helmholtz Zentrum München-German Research Center for Environmental Health, 85764 Neuherberg, Germany; Department of Medicine,§§ Internal Medicine, Lausanne University Hospital, 1015 Lausanne, Switzerland; Department of Clinical Physiology,‡‡ Turku University Hospital, 20520 Turku, Finland; Research Centre of Applied and Preventive Cardiovascular Medicine,|||| University of Turku, 20520 Turku, Finland; Department of Clinical Physiology,## Tampere University Hospital and University of Tampere, 33521 Tampere, Finland; German Centre for Cardiovascular Research (DZHK),$$ 80802 Munich, Germany; German Center for Diabetes Research (DZD e.V.),*** 85764 Neuherberg, Germany; Institute of Human Genetics,††† Technische Universität München, 81675 München, Germany; Munich Cluster for Systems Neurology (SyNergy),‡‡‡ 81377 Munich, Germany; Institute of Medical Informatics, Biometry, and Epidemiology,|||||| Ludwig-Maximilians-Universität, 81377 Munich, Germany; First Department of Internal Medicine,### Paracelsus Private Medical University, 5020 Salzburg, Austria; Cardiovascular Genetics Division,$$$ University of Utah School of Medicine, Salt Lake City, UT 84108; Department of Genetic Medicine,**** Weill Cornell Medicine, Doha, Qatar

**Keywords:** genetics, epidemiology, coronary artery disease

## Abstract

High lipoprotein (a) [Lp(a)] concentrations are an independent risk factor for cardiovascular outcomes. Concentrations are strongly influenced by apo(a) kringle IV repeat isoforms. We aimed to identify genetic loci associated with Lp(a) concentrations using data from five genome-wide association studies (n = 13,781). We identified 48 independent SNPs in the *LPA* and 1 SNP in the *APOE* gene region to be significantly associated with Lp(a) concentrations. We also adjusted for apo(a) isoforms to identify loci affecting Lp(a) levels independently from them, which resulted in 31 SNPs (30 in the *LPA*, 1 in the *APOE* gene region). Seven SNPs showed a genome-wide significant association with coronary artery disease (CAD) risk. A rare SNP (rs186696265; MAF ∼1%) showed the highest effect on Lp(a) and was also associated with increased risk of CAD (odds ratio = 1.73, *P* = 3.35 × 10^−30^). Median Lp(a) values increased from 2.1 to 91.1 mg/dl with increasing number of Lp(a)-increasing alleles. We found the *APOE2*-determining allele of rs7412 to be significantly associated with Lp(a) concentrations (*P* = 3.47 × 10^−10^). Each *APOE2* allele decreased Lp(a) by 3.34 mg/dl corresponding to ∼15% of the population’s mean values. Performing a gene-based test of association, including suspected Lp(a) receptors and regulators, resulted in one significant association of the *TLR2* gene with Lp(a) (*P* = 3.4 × 10^−4^). In summary, we identified a large number of independent SNPs in the *LPA* gene region, as well as the *APOE2* allele, to be significantly associated with Lp(a) concentrations.

High lipoprotein (a) [Lp(a)] concentrations have been shown to be an independent risk factor for CVD, as well as aortic valve calcification and stenosis. Mendelian randomization studies provided strong evidence for causality [for review see (1)]. Lp(a) is a lipoprotein consisting of a core LDL-like particle and the glycoprotein, apo(a), that are covalently linked to each other. It is a quantitative genetic trait under pronounced genetic control (1). Twin and family studies suggest that 90–95% of variation in Lp(a) is heritable (2). The distribution of Lp(a) concentrations in the population is extremely broad, with a more than 1,000-fold range from below 0.1 mg/dl to more than 200 mg/dl (1, 3). The concentration of Lp(a) is mostly influenced by the size of the apo(a) isoforms, which are caused by a coding copy-number-variation (CNV) in the *LPA* gene (2, 4, 5). This CNV contains 1 to >40 repeated plasminogen-like kringle IV (KIV) domains, the so-called KIV repeats, leading to a high heterogeneity of the apo(a) isoform distribution in populations. However, not all alleles are expressed (6), resulting in different heterozygosity rates on the DNA level (up to 95% heterozygosity) and protein level (up to 70% heterozygosity) (1). These nonexpressed alleles are not uniformly distributed: shorter isoforms are more likely to be expressed than longer isoforms and are associated with higher Lp(a) concentrations (1). Lp(a) belongs to the strongest genetically determined risk factors for CVD, considering that 25–35% of the population carry short isoforms that are usually associated with high Lp(a) concentrations and a doubling of the risk for CVD. Besides the number of KIV-2 domains, genetic variants in the broader *LPA* gene region, as well as other variants, have been found to be associated with Lp(a) concentrations (1). Fifty to ninety percent of the overall genetic variation in Lp(a) is attributable to the *LPA* locus (1).

So far, several genome-wide association studies (GWASs) on Lp(a) have been published (7–14). However, these studies were limited by small sample size (7, 12, 14), restricted scope due to the use of a specialized cardiovascular gene-chip (9, 10), or were conducted in certain subgroups, such as patients with type 2 diabetes (11), or in population isolates (8, 13). All of these studies primarily identified SNPs in the *LPA* gene cluster on chromosome 6q27 (*SLC22A3-LPAL2-LPA-PLG*). However, the SNPs with the highest effects were not independent from the KIV repeat (10). One recent GWAS in African Americans (14) also found one SNP in the *APOE* gene to be significantly associated with Lp(a) concentrations, which has been confirmed by a recent large study (15). However, Lp(a) levels and the types and frequencies of genetic variants, especially in the *LPA* gene, differ considerably between ethnic groups (3). Therefore, this finding is not generalizable to other populations.

Our project aimed to identify gene loci that are associated with Lp(a) concentrations on a hypothesis-free approach using genome-wide SNP chips in studies of European ancestry. We included adjustment for apo(a) isoforms to identify loci that affect Lp(a) levels independent from these apo(a) isoforms. The hypothesis-free approach, especially, is thought to provide new avenues to genes and thereby research directions to find answers to unresolved questions regarding physiology and pathophysiology, as well as production and catabolism of Lp(a). Besides the hypothesis-free approach, we performed a gene-based test of association for a list of possible candidate genes consisting of suspected Lp(a) receptors and regulators discussed in the literature. Altogether, five different primarily population-based studies with 13,781 individuals were included and about 10 million SNPs analyzed.

## MATERIALS AND METHODS

### Study design and description of cohorts

Genome-wide analyses were performed in five different studies individually and were then meta-analyzed. The genome-wide significance level was set to 5 × 10^−8^. One additional study (SAPHIR study) was included for an in-depth analysis of *APOE* genotypes. A detailed description of the cohorts is provided in the supplemental Materials and Methods and in supplemental Table S1. For information on genotyping and imputation see supplemental Table S2.

### Measurement of Lp(a) concentrations and apo(a) isoforms

For all participating studies, all Lp(a) measurements and apo(a) isoforms were performed by ELISA and immunoblot in the same laboratory (Division of Genetic Epidemiology, Innsbruck, Austria). Details on the measurement techniques are given in the supplemental Materials and Methods. A standardized amount of Lp(a) (150 ng) was loaded on the immunoblot. The predominantly expressed apo(a) isoform (in heterozygous samples), or the only band present (in homozygous samples) was used to adjust the statistical analysis for apo(a) isoforms. In individuals showing only one band in the Western blot, the second allele was either not expressed (16) or they were truly homozygous, although the proportion of individuals being truly homozygous on DNA level was expected to be lower than 5%.

### Statistical methods

#### GWAS analysis of single studies and meta-analysis.

Because the distribution of the Lp(a) concentrations were highly skewed, an inverse-normal transformation was applied to the measured Lp(a) concentrations. In each study, each SNP was tested for association with these inverse-normal transformed Lp(a) concentrations in an additive genetic model using linear regression, adjusting for age and sex (model 1). In addition, a second model, adjusted for age, sex, and the apo(a) isoform that was predominantly expressed in the immunoblot (model 2), was tested. To obtain interpretable effect estimates, linear regression was also performed on the original scale of Lp(a) for both models. Genome-wide analysis in the FamHS study was done using a linear mixed model accounting for familial dependencies described by a pedigree-based kinship matrix.

For the meta-analysis of all GWASs, the software, METASOFT (17), was used for all imputed SNPs that met imputation and quality control criteria and were present in at least two studies (∼9.2 M SNPs). Details on quality control, filtering criteria, and the meta-analysis approach are provided in the supplemental Materials and Methods and supplemental Fig. S1. Gender-stratified models were also applied for both models, followed by a *t*-test on effect differences between men and women (18). Pairwise linkage disequilibrium (LD) was evaluated using SNiPA with 1000 Genomes, phase1v3 data (19).

#### Conditional analysis.

To detect independently associated SNPs, a conditional stepwise analysis was performed using the program, GCTA [version 1.24.7 (20)]. For each locus with at least one *P* value <5 × 10^−8^, the SNP with the lowest *P* value was taken as the lead SNP. It was planned to include all SNPs within a region ±500 kb surrounding the lead SNP in the conditional analysis. Because genome-wide significant SNPs were also found outside of this range for the *LPA* gene region, the conditional analysis was extended to a range of 1.76 Mb. GCTA uses the summary statistics of the meta-analysis plus one reference population for LD calculation. As reference population, a combined genotype dataset of KORA F3 and KORA F4 was used (n = 6,002). By default, the lead SNP was included in the model first. Then, all SNPs in the included gene region were tested for association in addition to the already included SNPs in a stepwise manner. Using all independently associated SNPs from model 1, an unweighted, as well as weighted, SNP-score was derived. The unweighted SNP-score corresponded to the number of Lp(a)-increasing alleles. For weighting, β estimates on inverse-normal transformed Lp(a) values from the joint model of all included SNPs were taken.

#### Gene-based and candidate gene analysis.

In addition to the analysis of single SNP effects, a gene-based scan was performed using meta-analysis results from both models (with and without adjusting for isoforms) using the software, KGG version 3.5 (21). Gene regions were defined as the gene ±20 kb according to the RefGene database. Using this definition, 66.5% of all available SNPs were included. For the gene-based analysis, the extended Simes test (GATES) was used as implemented in KGG (22). To adjust for multiple testing, the Bonferroni method was applied on the number of tested genes (25,128 genes, which resulted in a significance level of 1.99 × 10^−6^). To calculate LD between the SNPs, the 1000G phase1v3 Reference was used. In addition to the hypothesis-free gene-based test, 21 candidate genes from literature were tested for association (supplemental Materials and Methods) using a Bonferroni significance *P* value of 0.05/21 = 0.0024.

#### Impact of Lp(a)-associated SNPs on coronary artery disease risk.

To assess the relevance of the identified SNPs with regard to risk on coronary artery disease (CAD), results from the CARDIoGRAMplusC4D consortium (23) were downloaded. This GWAS meta-analysis comprises studies of mainly European, but also South Asian and East Asian descent, including 60,801 CAD cases and 123,504 controls. Imputed genotypes were based on 1000 Genomes phase1v3. Log odds ratios (ORs) on CAD risk (assuming an additive model) and standard errors for all independently with Lp(a)-associated SNPs were retrieved from the summary-level data and matched to the minor allele.

#### Variance explained and heritability.

The combined dataset of both KORA studies (n = 6,002) was used to estimate the genomic heritability, which is the proportion of phenotypic variance explained by all tested SNPs (24). In addition, the proportion of variance explained by individual SNPs was calculated with data from both KORA studies using the software GCTA (v.1.24.7) (20). In the FamHS study, the proportion of the additive (polygenic) variance on the phenotypic variance, the narrow-sense heritability h^2^, was estimated using GenABEL’s polygenic function, taking the kinship matrix into account. This narrow-sense heritability thus also includes the variance explained by unmeasured SNP effects and other factors (e.g., CNVs).

#### Evaluating association of APOE genotypes with Lp(a) concentrations.

We followed up the identified association of a SNP in the *APOE* gene (rs7412) in both KORA studies and the SAPHIR study; the latter was not part of the GWAS meta-analysis. Two SNPs (rs7412 and rs429358) unambiguously defined the apoE isoforms (supplemental Table S3). Because *APOE* E2/E2 and E4/E4 genotypes are especially rather rare, a high imputation quality of at least 0.95 was required for both SNPs individually for this analysis. Therefore, imputed SNPs were only taken from the KORA F3 study, whereas de novo genotyping of these two SNPs was performed for KORA F4 and SAPHIR. The association analysis was performed for the *APOE* genotypes, E2/E2, E2/E3, E2/E4, E3/E4, and E4/E4, with the most common genotype, E3/E3, as the reference, adjusted for age and sex and also age, sex, and apo(a) isoforms. All three datasets (KORA F3, KORA F4, and SAPHIR) were combined in one dataset and mixed effect models were performed both on inverse-normal transformed Lp(a) levels and on the original scale of Lp(a).

### Bioinformatic analysis

In order to shed some light on potential functional elements underlying the identified SNPs, all of our GWAS hits, as well as all SNPs correlated with them (r^2^ ≥ 0.8), were investigated for: *1*) being a coding SNP or being located at a canonical splice site; *2*) being located in a transcription factor binding site reported by ENCODE (25); *3*) being located in a DNase hypersensitive site reported by ENCODE; or *4*) being located in a validated regulatory element reported by ORegAnno (26). GTex (27) was searched for reported eQTL SNPs for *LPA* (i.e., SNPs being correlated with *LPA* expression). Detailed methods are described in the supplemental Materials and Methods.

## RESULTS

### Description of cohorts and quality control

Supplemental Table S1 gives the descriptive characteristics of all contributing studies. The P-Z-plots did not reveal any deviations of the reported *P* values and the *P* values calculated by the β coefficient and standard error. The genomic inflation factor λ ranged from 1.011 to 1.030 (supplemental Table S2).

### Results of meta-analysis

**Fig. 1.**
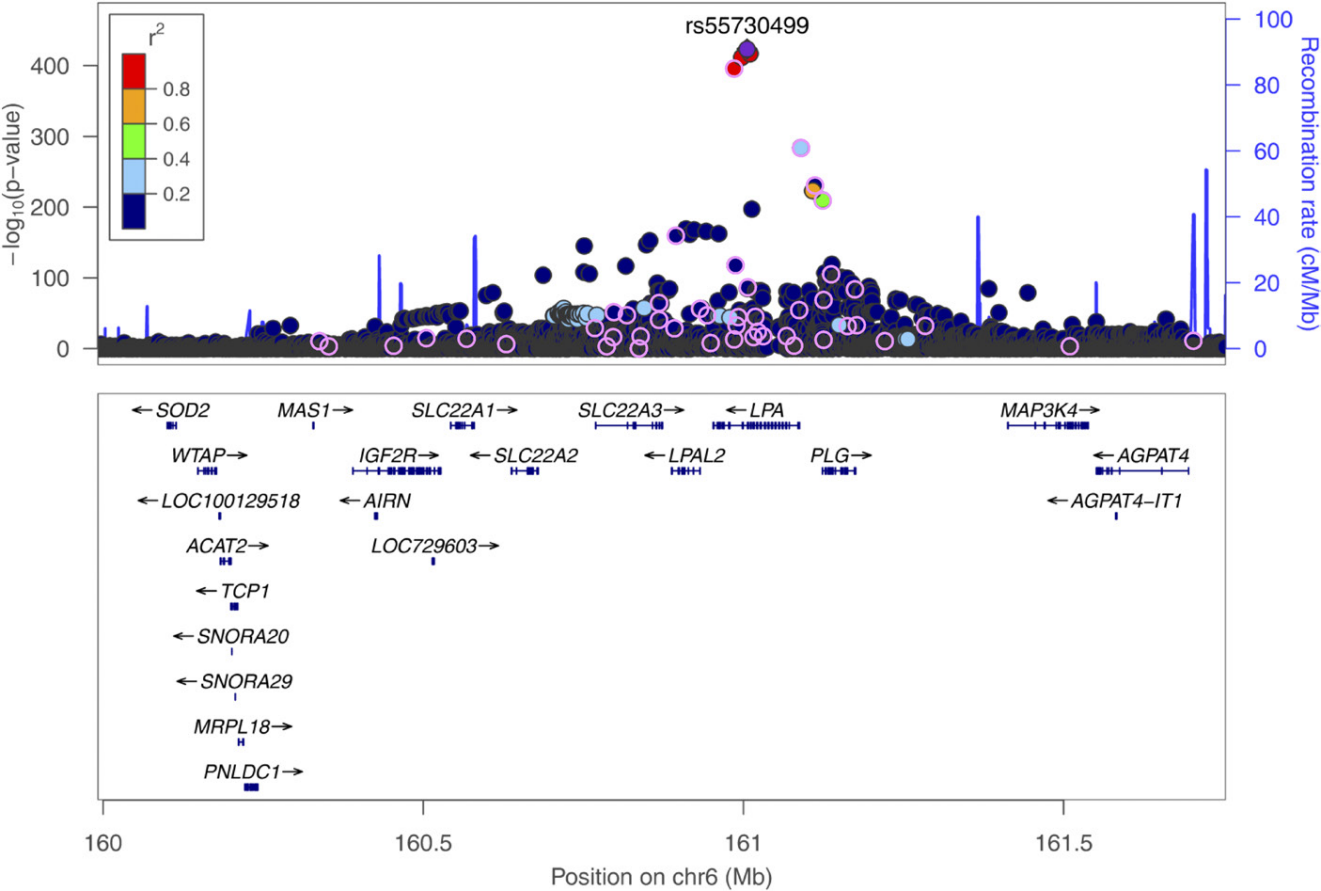
Regional plot showing the genomic region around the *LPA* gene (chr6:159,991,850-161,753,083; LD refers to rs55730499, based on 1000G EUR); *P* values are derived from the meta-analysis on the five cohorts including 13,781 individuals on inverse-normal transformed Lp(a) concentrations, adjusted for age and sex. All 48 SNPs, which are independently associated with Lp(a) in a joint model, are circled.

**Fig. 2.**
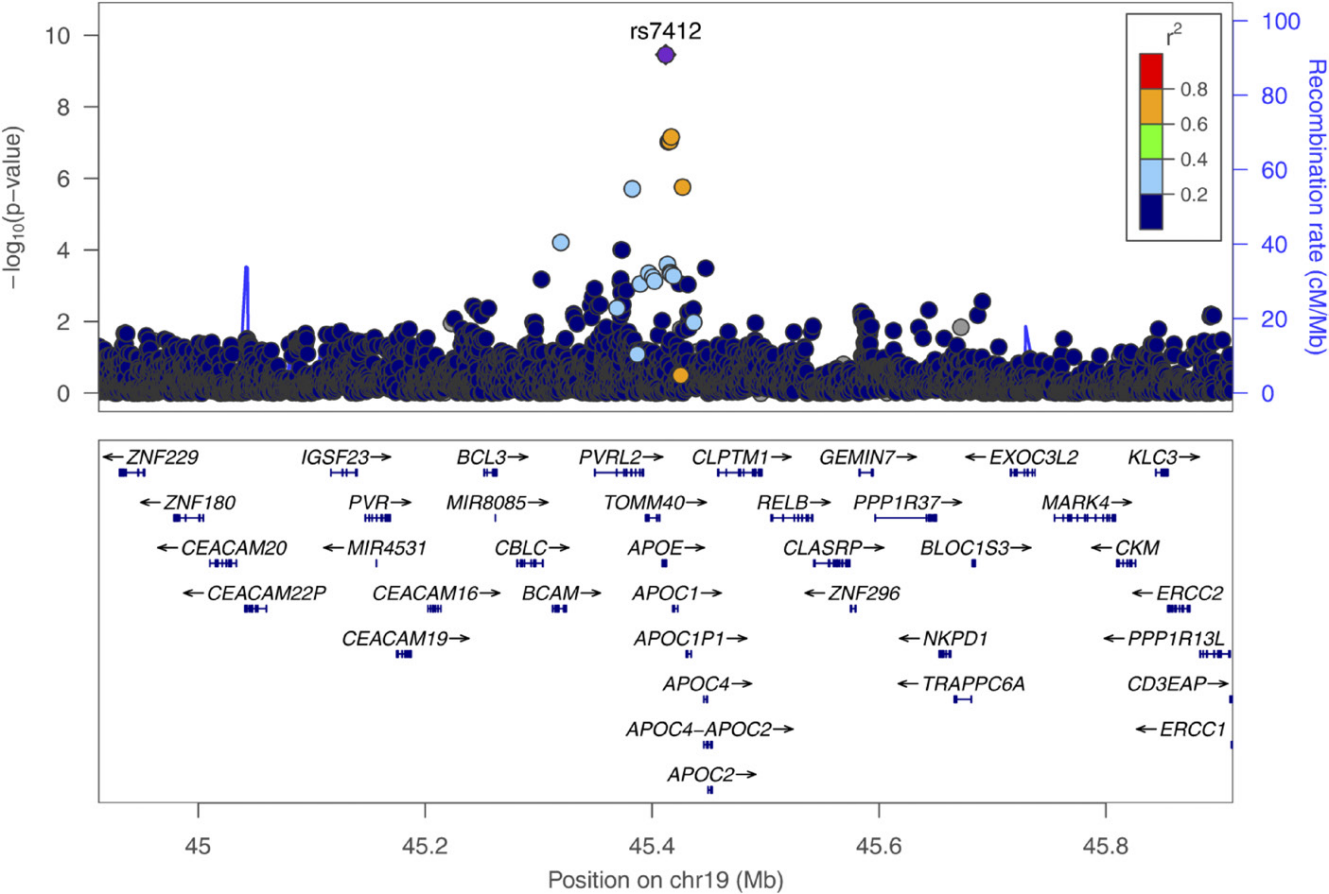
Regional plot showing the genomic region defined by the *APOE* lead SNP, rs7412, ±500 kb (LD refers to rs7412, based on 1000G EUR); *P* values are derived from the meta-analysis on the five cohorts on inverse-normal transformed Lp(a) concentrations, adjusted for age and sex.

In both models (with and without adjusting for the predominantly expressed apo(a) isoform), SNPs in two gene regions were identified: *LPA* and *APOE*. Manhattan plots for both meta-analyses are shown in supplemental Figs. S2 and S3 and corresponding QQ-plots in supplemental Figs. S4 and S5. On chromosome 6, surrounding the *LPA* gene, 2,001 SNPs reached genome-wide significance in model 1, with the lowest *P* value for SNP rs55730499 (*P* = 3.6 × 10^−424^, **Fig. 1**), and 1,961 SNPs reached genome-wide significance in model 2, with the lowest *P* value for SNP rs75692336 (*P* = 2.90 × 10^−216^, supplemental Fig. S6). Genome-wide significant SNPs for both models on chromosome 6 were scattered over a broad region spanning 1.76 Mb (chr6:159,991,850-161,753,083). Additionally, in the *APOE* gene on chromosome 19, one genome-wide significant SNP (rs7412) was identified in model 1 (*P* = 3.47 × 10^−10^, **Fig. 2**), and three genome-wide significant SNPs in model 2 (lowest *P* value for SNP rs7412: 3.48 × 10^−9^; supplemental Fig. S7).

### Further evaluation of the *LPA* gene region

The conditional analyses were performed for both models, including all SNPs in the 1.76 Mb-spanning broad *LPA* gene region. Forty-eight SNPs were independently associated with inverse-normal transformed Lp(a) in model 1 (see supplemental Table S4 for characteristics of SNPs, supplemental Table S5 for results of the single studies, and supplemental Table S6 for results of meta-analysis). These SNPs were primarily located within the *LPA* gene, but they were also scattered widely in a broad region up to ∼1 Mb away from the *LPA* gene (Fig. 1). The effect sizes on the original scale of Lp(a) ranged between 0.05 and 64.74 mg/dl per allele for the single SNPs and from 1.55 to 47.60 mg/dl per allele in a joint model including all 48 SNPs (median effect size per SNP: 5 mg/dl).

Thirty SNPs remained statistically significant after conditional stepwise analysis in model 2 (see supplemental Table S4 for characteristics of SNPs, supplemental Table S7 for results of the single studies, and supplemental Table S8 for results of meta-analysis). Again, these SNPs were distributed over the entire broad *LPA* gene region, most of them in or close to the *LPA* gene. Nineteen SNPs were unique to this isoform-adjusted model and were not included in the model where we did not adjust for the apo(a) isoforms (supplemental Table S4). However, three of them were in LD (r^2^ > 0.8) with SNPs from model 1 (rs140570886 in LD with rs1510225 from model 1 and rs3798220; rs55730499 in LD with rs118039278 from model 1 and rs10466872; rs59614420 in LD with rs4252109 from model 1). Therefore, 16 SNPs, which were identified in model 1, were associated with Lp(a) independently from isoforms and other Lp(a)-associated SNPs.

**Fig. 3.**
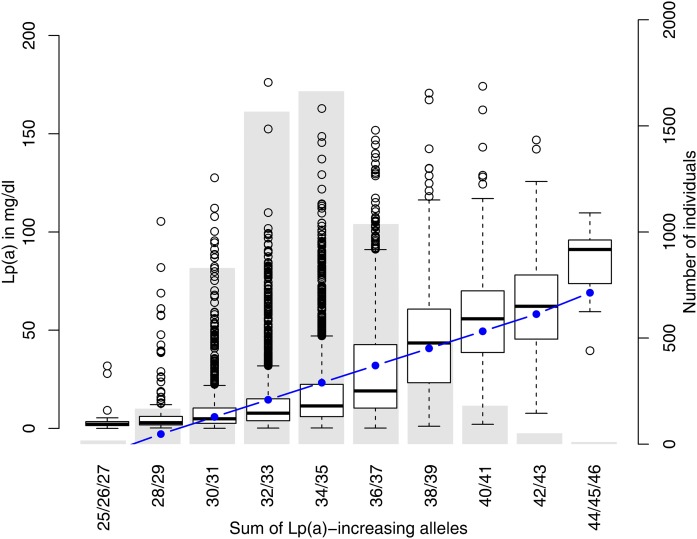
Boxplot of Lp(a) concentration for groups of a SNP-score [sum of Lp(a)-increasing alleles] derived from the 48 independent SNPs in the broad *LPA* gene region. An underlying bar plot shows the distribution of the score in KORA F3 and KORA F4. The blue line indicates the predicted values of Lp(a) for mid-interval values of the SNP-score, based on a linear regression from the SNP-score on Lp(a).

Both weighted and unweighted SNP-scores were derived from all 48 SNPs in the conditional model 1. **Figure 3** shows that median values of Lp(a) increased from 2.1 mg/dl for individuals with the minimum number of Lp(a)-increasing alleles to 91.1 mg/dl for individuals with the maximum number of Lp(a)-increasing alleles. A linear increase was observed, but in the medium range of the risk score many outliers were located at the upper tail of the Lp(a) distribution. This might be due to the skewed distribution of Lp(a) concentrations and the very wide range of the singular SNP effects (see above). Nevertheless, the weighted SNP-score explained 36% of the phenotypic variance of Lp(a) concentrations.

### Evaluation of potential functional or regulatory impact

We first investigated to determine whether the marker SNP or any of its proxies were known coding variants (supplemental Tables S4, S9). This resulted in 12 SNPs being located in exons, 6 thereof being missense variants and 6 being synonymous variants. Interestingly, all six missense variants were located in *LPA*, while only one of the six synonymous variants was located in *LPA*. The rs41272110 (*LPA* KIV-8), rs41259144 (*LPA* KIV-4), and rs41267807 (*LPA*, protease domain) were concordantly reported as being damaging by Polyphen (28) and SIFT (29), whereas the well-known *LPA* SNP, rs3798220, was classified as benign by SIFT, but as “possibly damaging” by Polyphen. The two remaining missense SNPs, rs4252125 in *PLG* and rs41267809 in *LPA*, were concordantly predicted to be benign. The previously described SNP, rs41272114, is located on a splice site and causes null alleles (30–32). Other known nonsense variants are too rare (33) to be detected by GWASs or occur in different ethnicities (34).

However, additional in silico evaluation of these SNPs was hampered by the fact that GTEx, the largest eQTL resource available so far, does not yet report eQTL data for *LPA* in liver tissue, while *LPA* expression is tightly regulated and happens nearly exclusively in liver tissue.

### Further evaluation of the *APOE* gene region

**Fig. 4.**
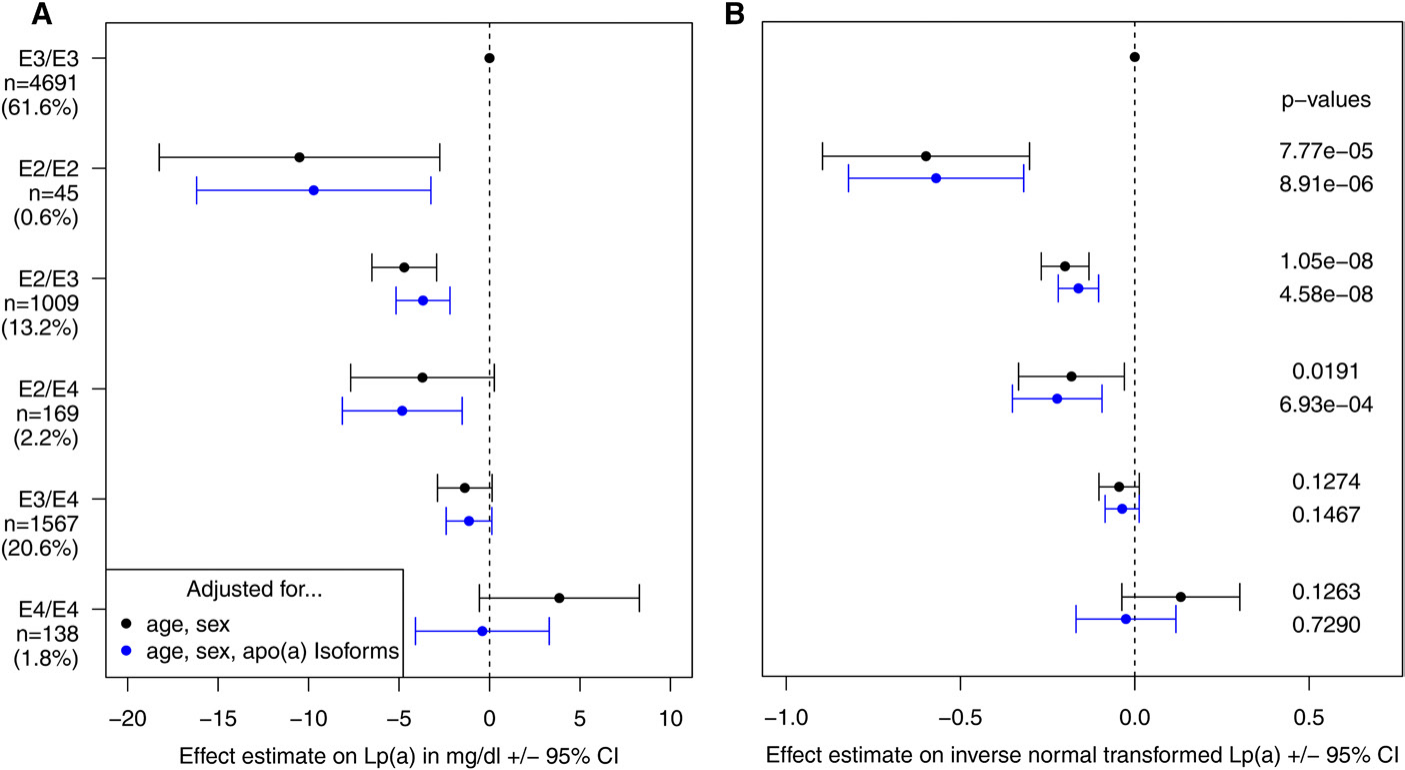
Results of a mixed model (using data from KORA F3, KORA F4, and SAPHIR combined) evaluating the effects from *APOE* genotypes, defined as described in supplemental Table S3, on untransformed Lp(a) values in milligrams per deciliter (A), as well as on inverse-normal transformed Lp(a) (B). Both panels show β estimates and 95% CI for age- and sex-adjusted models (in black), as well as age-, sex-, and isoform-adjusted models (in blue). *P* values are derived from the model using inverse-normal transformed Lp(a).

Conditional analyses, including SNPs from the *APOE* region (lead SNP ±500 kb: chr19: 44,912,079-45,912,079) yielded no additional genome-wide significant SNPs after adjusting for the top SNP (rs7412). This SNP defined the *APOE2* allele and explained 0.5% of the phenotypic variance of inverse-normal transformed Lp(a) levels. Each *APOE2* copy decreased Lp(a) concentrations by 3.34 mg/dl corresponding to ∼15% of the population’s mean values (supplemental Table S6). An extended evaluation of APOE genotypes was performed using data from the KORA F3, KORA F4, and the SAPHIR studies combined. The E2/E2 genotype was associated with a decrease of Lp(a) by 10.5 mg/dl compared with the E3/E3 genotype (*P* = 7.77 × 10^−5^), while the effect was about half as high (4.7 mg/dl) for E2/E3 (*P* = 1.05 × 10^−8^) and E2/E4 (*P* = 0.0191), indicating a rather additive effect of the E2 allele (**Fig. 4**). Conversely, E4/E4 was associated with a nonsignificant increase of Lp(a). Further adjusting for apo(a) isoforms only attenuated the effect estimates for E4/E4, but did not change the effect estimates and/or *P* values for the other *APOE* genotypes.

### Gender-stratified analyses

GWASs stratified for gender did not result in any additional genome-wide significant hits outside the wider *LPA* and *APOE* gene region, neither for men, nor for women. There was also no genome-wide significant SNP-gender interaction effect for both adjustment models.

### Genome-wide variance explained and heritability

The genome-wide SNP-based explained variance, including the entire dataset of available SNPs (genomic heritability), was estimated to be 49.3% in both KORA studies [95% CI: (32.0%; 66.5%)]. A narrow-sense heritability h^2^ of Lp(a) was calculated to be 91.7% from the polygenic model in the family-based FamHS study. This estimate included not only the measured SNP effects, but also unmeasured factors.

### Gene-based and candidate gene analyses

The genome-wide gene-based association scan resulted in 23 significant genes for the inverse-normal transformed Lp(a) concentrations in model 1 and in 20 significant genes in model 2. All of them were located either in the broad *LPA* or *APOE* gene region (supplemental Table S10).

The gene-based test of association for a list of possible candidate genes consisting of suspected Lp(a) receptors and regulators discussed in the literature resulted in one significant association of the *TLR2* gene with Lp(a) for model 1 (*P* = 3.4 × 10^−4^; supplemental Table S11). No significant association within the candidate genes was found for model 2 (supplemental Table S11).

### Impact of Lp(a)-associated SNPs on CAD risk

**Fig. 5.**
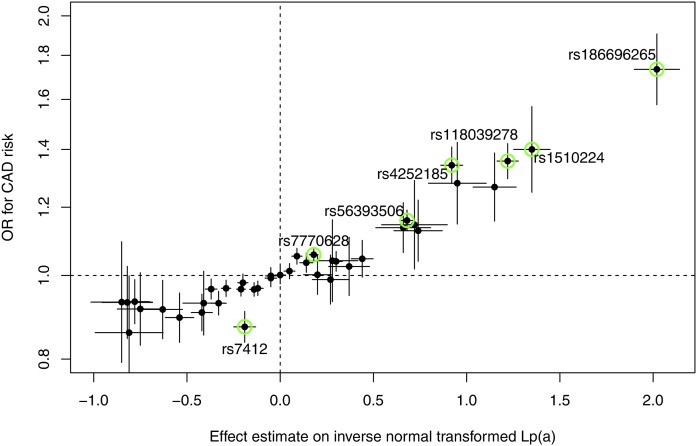
Scatterplot showing the effect estimates on inverse normally transformed Lp(a) levels (±95% CI) on the x axis and the ORs for CAD risk (±95% CI) on the y axis (derived from theCARDIoGRAMplusC4D consortium) for all 40 SNPs, which were identified in model 1 (age- and sex-adjusted) and which were available in the CARDIoGRAMplusC4D results. All SNPs, which are also genome-wide significantly associated with CAD risk, are marked in green.

From all 49 genetic variants that were shown to be independently associated with Lp(a) in model 1 (48 in the *LPA* gene region plus rs7412 in *APOE*), 40 were present in summary-level data retrieved from the CARDIoGRAMplusC4D consortium. Nine low-frequency variants (MAF <1%) were missing. **Figure 5** shows how the effect estimates on Lp(a) relate to the ORs for CAD risk. Seven SNPs were even significantly associated with CAD on a genome-wide scale (*P* < 5 × 10^−8^). The highest effect was for rs186696265, which showed an OR of 1.73 (*P* = 3.35 × 10^−30^) with CAD risk for each copy of the minor allele (supplemental Table S13).

## DISCUSSION

The meta-analysis of five different studies revealed SNPs in two genomic regions to be significantly associated with Lp(a) concentrations. Within a broad region surrounding the *LPA* gene, about 2,000 genome-wide significant SNPs were identified, of which 48 were still genome-wide significant in a joint model after stepwise conditional analysis. The apo(a) isoforms determined via Western blot were available for all 13,781 participants included in the meta-analysis. To our knowledge, this is the largest GWAS meta-analysis on Lp(a) concentrations adjusting for apo(a) isoforms. Adjusting for apo(a) isoforms resulted in 30 independently associated SNPs in the broad *LPA* gene region, most of them different from the SNPs found in the previous model. In addition, we found that carriers of the *APOE2* allele of the *APOE* locus had significantly lower Lp(a) levels compared with *APOE* E3/E3 genotypes. The clinical impact of the SNPs found to be associated with Lp(a) concentrations was underscored by the observation that seven SNPs were even significantly associated with CAD on a genome-wide scale in the CARDIoGRAMplusC4D consortium.

### Findings in the *LPA* gene region

The largest contributor of Lp(a) concentration variability was the number of the KIV repeats in the *LPA* gene. Furthermore, several SNPs in the *LPA* gene region were found to be associated with Lp(a) concentrations. For many of the associations, it was not clear whether they causally regulate Lp(a) concentrations, as discussed recently (1). However, the leading SNPs in GWASs on Lp(a) have not been such functional SNPs anyway, but rather SNPs correlated with KIV repeats. Two SNPs have been repeatedly reported and are already well-established: rs10455872 and rs3798220 (9–11, 13). Both SNPs have been found to partially tag short apo(a) isoforms with 17–20 and 19–21 repeats, respectively. Therefore, carriers of the minor alleles of these two SNPs have higher Lp(a) levels and consequently higher risk of developing coronary disease (10). However, only about half of the short high-risk apo(a) isoforms are tagged by these two SNPs (35). Moreover, rs3798220 has been reported to be associated with increased oxidized phospholipid (OxPL) carriage on apoB-100 (36, 37).

The 48 SNPs that remained significant after applying a stepwise conditional model can be seen as rather independent from each other and explain 36% of the variance in Lp(a) concentrations. The lead SNP, which was selected in the conditional analysis (rs118039278) was in perfect LD (r^2^ = 1) with the well-known KIV-tagging SNP, rs10455872 (*P* = 9.7 × 10^−418^). The other KIV-tagging SNP, rs3798220, was not included in the 48 SNP-score, but was captured by another SNP in perfect LD (rs1510224). Three out of the 48 SNPs have already been described in the literature: rs7770628 has been found to be associated with Lp(a) concentrations greater than 14 mg/dl (7); rs41272114 causes a splicing defect and results in a null-allele (38), and rs186696265 has been identified in two recent GWAS meta-analyses on lipid-traits aiming to identify specifically rare variants in already known lipid loci (39, 40). The SNP, rs186696265, had a MAF of 1.1% (1000G phase1v3) and was shown to be associated with LDL cholesterol (LDL-C) and total cholesterol, but only in Europeans. In our analysis, this SNP had the highest effect size on Lp(a) concentrations; each copy of the minor allele of this SNP increased Lp(a) levels by 65 mg/dl, or by 48 mg/dl in a joint model after adjustment for all of the 47 other independent SNPs. As Lp(a) particles contain roughly 30% cholesterol (41) that is included in each common cholesterol measurement method, the recently found association of this SNP with LDL-C and total cholesterol (39, 40) might be explained by the effect of this SNP on Lp(a) concentrations. An Lp(a)-increasing effect of 65 mg/dl would translate into an increase of LDL-C of ∼20 mg/dl. Of note, this SNP is located between two intergenic enhancer regions, which have been proposed to regulate *LPA* expression (42) (supplemental Fig. S8) and is in partial LD (r^2^ = 0.69, D′ = 0.95) with a SNP within the putative regulatory enhancer region, DHIII (rs7758766) (43). This SNP also shows a partial LD (r^2^ = 0.64) with rs3798220, described by Clarke et al. (10).

To detect SNPs whose effects did not merely reflect LD with the KIV repeat, we used models additionally adjusting for apo(a) isoforms. Former studies looking at correlations of SNPs in the *LPA* gene region with KIV repeats or isoform size led to inconsistent results: one study found no correlation of SNPs with KIV repeats (44), while others concordantly found a high correlation of one specific SNP (rs10455872) with KIV copy number and short isoform (10, 45). The largest GWAS up to now that adjusted for apo(a) isoforms was performed in 1,376 Old Order Amish (13). However, only SNPs on chromosome 6q25-26 were adjusted for isoforms, which resulted in a modest reduction of the strength of the SNPs in this region with the Lp(a) cholesterol levels. As the published results are heterogeneous, we concluded that a systematic adjustment for the apo(a) isoforms on a large scale was needed to: *1*) disentangle the influence of partially correlated variants on Lp(a) levels with the *LPA* gene region; and *2*) increase power for detecting other genes that have an influence on Lp(a) levels. However, adjusting for apo(a) isoforms is not straightforward. The analysis of isoforms on the protein level by Western blot provides allele-specific isoform sizes. Because not all apo(a) isoforms are expressed, the detected apo(a) isoforms do not fully reflect the alleles on the DNA level. Furthermore, even if both isoforms can be detected, one of them, usually the shorter one, might be predominating in plasma. From several different statistical modeling strategies, the predominantly expressed apo(a) isoform explains the highest variability of Lp(a) and is, in our view, the best way to account for apo(a) isoforms in statistical models. Still, the contribution of the second isoform is ignored and therefore, adjusting for the predominantly expressed apo(a) isoform can only partly remove the association with SNPs that is just due to LD with KIV repeats.

Consequently, known KIV-tagging SNPs (rs10455872, rs3798220) were still highly significantly associated with Lp(a) concentrations after adjusting for apo(a) isoforms, but the association was attenuated massively. In this isoform-adjusted model, a set of 30 SNPs independently associated with Lp(a) was identified by using a stepwise conditional model, 16 of them unique and not correlated with any SNP contained in model 1. The top-hit (rs75692336) represented a cluster of correlated SNPs spanning over the *LPA* gene, the intergenic region between *LPA* and *PLG*, and the *PLG* gene. It was highly correlated with the missense variant, rs41272110, in KIV-8 [r^2^ = 0.87; also known as T23P (30) or T12P (46)], which was predicted to be deleterious to the protein function and has been associated with reduced Lp(a) levels before (30, 46). Moreover, we recently found rs75692336 to tag a frequent splice-site variant within the KIV-2 repeat, that explains 20% of the Lp(a) variance in low molecular weight isoform carriers (47). This splice-site variant was discovered by means of a next generation ultra-deep sequencing approach, which aimed to explain Lp(a) values that were discordant to what one would expect from the respective apo(a) isoforms. In the same manner, our isoform-adjustment approach seemed to find not only isoform-independent SNPs, but particularly SNPs leading to Lp(a) values that deviated from what would be expected given the observed isoform. As in the first model, the highest effect size on Lp(a) concentrations was found for SNP rs186696265, located near the known intergenic enhancer regions (supplemental Fig. S8). Two out of the 16 unique and independently associated SNPs were already described in the literature. The SNP, rs3798221, was already found to be associated with Lp(a) levels (10), MI (48), Lp(a) levels in women (49), and was found to be associated with Lp(a) concentration and marginally associated with KIV copy number in three ethnicities (South Asians, Chinese, and European Caucasians) (45). The SNP, rs56393506, was already found the be associated with Lp(a) levels (50). Because these 16 independent SNPs were only identified by adjusting for the apo(a) isoform, they might exert an effect on Lp(a) and, as a result, on CAD risk only in subgroups of isoforms (e.g., only in low or high molecular weight isoforms), which should be subject to further research.

### Association with *APOE*

Previous studies evaluating the association of variants in the *APOE* gene with Lp(a) were mostly non-GWASs and produced inconsistent results (supplemental Table S12). While some reported no contribution of *APOE* to Lp(a) variation, others showed an Lp(a)-lowering effect of *APOE2* compared with *APOE3*, partially in a context-dependent manner, e.g., in women or apo(a) isoform dependent. We clearly show that the Lp(a)-lowering effect of *APOE2* did not depend on either gender or apo(a) isoform size.

apoE is part of chylomicrons, remnants, and VLDL, IDL, and HDL particles and mediates removal of remnants from circulation via *LDLR*, *LRP1*, and *HSPG* receptors (51). apoE has also been reported in a triglyceride-rich subspecies of circulating Lp(a) particles (52). Two missense SNPs (rs7412, rs429358) define three isoforms named E2, E3, and E4, the two mutant isoforms E2 and E4 presenting markedly different physiological functions (51). E2 presents only ∼1% of the LDLR binding affinity compared with E3 and E4 (51) and results in impaired removal of VLDL particles (53). Coincidence of homozygous *APOE2* and conditions causing high VLDL, such as obesity, led to hyperlipoproteinemia type III (54).

The observation that *APOE2* was associated with lower Lp(a) levels confirms previous reports (15, 55), albeit the mechanism has not been elucidated yet. Effects of the *APOE* genotype on both Lp(a) catabolism and synthesis have been proposed (15). Assuming a competition of LDL-C, Lp(a), and apoE-carrying triglyceride-rich lipoproteins for common receptors, it has been suggested recently that Lp(a) concentrations are decreased in *APOE2* carriers because the decreased receptor affinity of apoE2 increases the number of receptors available for LDL-C and Lp(a) (15). On the other hand, an effect on synthesis rate is also conceivable. *APOE* genotypes exert several secondary effects on lipoprotein metabolism by altering the hepatic lipoprotein remnant metabolism (55). Given that Lp(a) is synthesized by the liver (3) and synthesis rate represents the main determinant of Lp(a) levels (56), *APOE2* may thus affect the availability of substrate for Lp(a) formation (55).

### Gene-based candidate gene analyses

To investigate minor effects not captured by a single-SNP analysis, we performed a gene-based analysis for candidate genes that were reported to bind Lp(a) and/or regulate *LPA* or *PCSK9*. Finally, only *TLR2* showed a significant association with Lp(a) levels. *TLR2* has been shown to concur with *CD36* and *TLR6* in Lp(a)-transported OxPL (57). However, to our knowledge, the reverse effect of an influence of *TLR2* on Lp(a) levels has not been shown yet. Because neither scavenger receptor *CD36*, which internalizes OxPL, nor *TLR6*, which dimerizes with *TLR2* in OxPL recognition (57), were significant in our analysis; the biological basis of this association might instead reside in the other manifold roles of *TLR2* in innate immunity and inflammation (58). Acute phase reactions have been proposed to affect Lp(a) levels, albeit the magnitude and direction remains controversial (1). Furthermore, in mice, *TLR2* activation was also shown to result in a ∼14-fold increase in *PCSK9* expression (59), another known regulator of Lp(a) in humans. Thus various potential routes of action of *TLR2* on Lp(a) levels exist, although strict replication and further studies will be needed to elucidate the mechanism of this finding.

### Heritability and explained variance of Lp(a) concentrations

The genome-wide-SNP-based explained variance, including the entire dataset of available SNPs (genomic heritability), was estimated to be 49.3% in both KORA studies. In contrast, the narrow-sense heritability h^2^ of Lp(a) derived from the polygenic model in the family-based FamHS study, was estimated to be 91.7%. The genomic heritability only includes the measured SNP effects. The narrow-sense heritability also includes unmeasured SNP effects, CNVs, and other genetic effects. A major reason for the big difference between these two heritability estimates is most probably due to the KIV repeats, which are only partly covered by LD with the measured SNP effects.

The weighted SNP-score that was derived based on the 48 independently associated SNPs from model 1 explained 36% of the phenotypic variance of Lp(a) concentrations. As two SNPs of the SNP-score (rs118039278 and rs1510224) were in perfect LD with the SNPs, rs10455872 and rs3798220, partially tagging KIV, this estimate of explained variance might also be influenced by effects from the KIV repeats and not solely from the SNPs themselves. These two SNPs, which are usually taken as genetic instruments to predict Lp(a), jointly explain roughly 20% of the phenotypic variance of Lp(a) in the KORA studies we used for our meta-analyses. Therefore, we would expect an improvement in accuracy in analyses containing a SNP score when taking the 48 SNPs instead of taking rs10455872 and rs3798220 only.

### Impact of Lp(a)-associated SNPs on CAD risk

We observed a direct proportional relationship of Lp(a)-associated variants with CAD risk increase for Lp(a)-increasing variants and a decrease in risk for Lp(a)-decreasing variants. This is in line with several previous studies, which were performed primarily on the highly cited variants, rs10455872, rs3798220, and rs41272114 (10, 60, 61). In pre­vious studies that evaluated the causal relationship of Lp(a) with CAD, rs10455872 and rs3798220, especially, have been used as instrumental variables to determine the “genetically regulated” proportion of Lp(a). Although both SNPs are not included in our identified SNP set, they are replaced by other variants (rs118039278 and rs3798220) in perfect LD, which are associated with an OR for CAD risk of 1.36 and 1.40, respectively. We could also show that the SNP, rs186696265, which presents the highest effect size on Lp(a) in our investigation, also presents the highest OR for CAD of all investigated SNPs (OR = 1.73) in the *LPA* gene region. Therefore, with respect to CAD risk, rs186696265 seems to be at least equivalent or even superior to the well-known SNPs. Because this SNP is rather rare, it has not been detected before.

The exact functional effects of the SNPs annotated in Fig. 5 on CAD risk are still unknown and might reside both in the regulation of Lp(a) levels or, in the case of the rs3798220-tagging SNP, rs1510224, in modification of OxPL carriage on apoB100 (36). It is also remarkable that only one SNP seems to deviate from the linear relationship between effect sizes on Lp(a) and the log ORs for CAD risk, which is rs7412. Because rs7412 in the *APOE* gene is also associated with a decrease in LDL-C, independently from its association on Lp(a), the effect on CAD risk is not only due to Lp(a), but also might be triggered by a reduction in LDL-C.

Altogether, our findings add to the growing evidence that lowering Lp(a) levels might be a valuable strategy to reduce the risk of CAD (61). This is even more important in the light of emerging therapies, such as PCSK9-inhibitors or specific oligonucleotide therapies, that are able to lower Lp(a) levels by up to 30% and 90%, respectively (62, 63).

## CONCLUSIONS

This GWAS meta-analysis in 13,781 participants from five different cohorts revealed up to 48 independent SNPs in the broad *LPA* gene region that are associated with Lp(a). One rather rare variant, which was shown to be associated with Lp(a) independently from apo(a) isoforms, was associated with the highest effect size on Lp(a) of all investigated SNPs and likewise also with the highest CAD risk. This investigation also provided evidence of the involvement of the apoE2 isoform, as well as the *TLR2* gene, in the regulation of Lp(a) concentrations.

## Supplementary Material

Supplemental Data
