# Supplementary material for: A genome-wide association meta-analysis on lipoprotein (a) concentrations adjusted for apolipoprotein (a) isoforms
Source: J Lipid Res. 2017 May 16;58(9):1834–44. doi: 10.1194/jlr.M076232 (PMC5580897; doi:10.1194/jlr.M076232)
Supplement: Supplemental Data [file supp_58_9_1834__index.html]

A genome-wide association meta-analysis on lipoprotein (a) concentrations adjusted for apolipoprotein (a) isoforms — Supplemental Data 

# A genome-wide association meta-analysis on lipoprotein (a) concentrations adjusted for apolipoprotein (a) isoforms

## Supplemental Data

- Supplementary (.pdf, 959 KB) - Supplementary Material
