# Supplementary material for: A genome-wide association meta-analysis on lipoprotein (a) concentrations adjusted for apolipoprotein (a) isoforms
Source: J Lipid Res. 2017 May 16;58(9):1834–44. doi: 10.1194/jlr.M076232 (PMC5580897; doi:10.1194/jlr.M076232)
Supplement: Supplemental Data [file 10.1194_M076232_jlr.M076232-1.pdf]

Supplementary Material to

A genome-wide association meta-analysis on lipoprotein(a) concentrations adjusted for apolipoprotein(a) isoforms

Salome Mack[1], Stefan Coassin[1], Rico Rueedi[2,3], Noha A. Yousri[4,5], Ilkka Seppälä[6], Christian Gieger[7,8,9], Sebastian Schönherr[1], Lukas Forer[1], Gertraud Erhart[1], Pedro Marques-Vidal[10], Janina Ried[7], Gerard Waeber[10], Sven Bergmann[2,3], Doreen Dähnhardt[1], Andrea Stöckl[1], Olli T Raitakari[11,12], Mika Kähönen[13], Annette Peters[8,14,15], Thomas Meitinger[16,17,18], Konstantin Strauch[7,19], KORA-Study Group, Ludmilla Kedenko[20], Bernhard Paulweber[20], Terho Lehtimäki[6], Steven C. Hunt[21,22], Peter Vollenweider[10], Claudia Lamina[1,23,§,*], Florian Kronenberg[1,23,§,*]

[1] Division of Genetic Epidemiology, Department of Medical Genetics, Molecular and Clinical Pharmacology, Medical University of Innsbruck, 6020 Innsbruck, Austria

[2] Department of Computational Biology, University of Lausanne, 1015 Lausanne, Switzerland

[3] Swiss Institute of Bioinformatics, 1015 Lausanne, Switzerland

[4] Department of Physiology and Biophysics, Weill Cornell Medical College − Qatar, Doha, Qatar

[5] Department of Computer and Systems Engineering, Alexandria University, 21526 Alexandria, Egypt

[6] Department of Clinical Chemistry, Fimlab Laboratories and University of Tampere School of Medicine, 33520 Tampere, Finland

[7] Institute of Genetic Epidemiology, Helmholtz Zentrum München—German Research Center for Environmental Health, 85764 Neuherberg, Germany

[8] Institute of Epidemiology II, Helmholtz Zentrum München—German Research Center for Environmental Health, 85764 Neuherberg, Germany

[9] Research Unit of Molecular Epidemiology, Helmholtz Zentrum München—German Research Center for Environmental Health, 85764 Neuherberg, Germany

[10] Department of Medicine, Internal Medicine, Lausanne University Hospital, 1015 Lausanne, Switzerland

[11] Department of Clinical Physiology, Turku University Hospital, 20520 Turku, Finland

[12] Research Centre of Applied and Preventive Cardiovascular Medicine, University of Turku, 20520 Turku, Finland

[13] Department of Clinical Physiology, Tampere University Hospital and University of Tampere, 33521 Tampere, Finland

[14] DZHK (German Centre for Cardiovascular Research), partner site Munich Heart Alliance, 80802 Munich, Germany

[15] German Center for Diabetes Research (DZD e.V.), 85764 Neuherberg, Germany

[16] Institute of Human Genetics, Technische Universität München, 81675 München, Germany

[17] Institute of Human Genetics, Helmholtz Zentrum München, 85764 Neuherberg, Germany

[18] Munich Cluster for Systems Neurology (SyNergy), 81377 Munich, Germany

[19] Institute of Medical Informatics, Biometry and Epidemiology, Chair of Genetic Epidemiology, Ludwig-Maximilians-Universität, 81377 Munich, Germany

[20] First Department of Internal Medicine, Paracelsus Private Medical University, 5020 Salzburg, Austria

[21] Cardiovascular Genetics Division, University of Utah School of Medicine, Salt Lake City, UT 84108, USA

[22] Department of Genetic Medicine, Weill Cornell Medicine, Doha, Qatar

[§] **These authors contributed equally to this work**

***Address of correspondence:**

Florian Kronenberg
Division of Genetic Epidemiology
Department of Medical Genetics, Molecular and Clinical Pharmacology
Medical University of Innsbruck
Schöpfstr. 41, A-6020 Innsbruck, AUSTRIA
Phone: (+43) 512 9003-70560
Fax: (+43) 512 9003-73560 or -73561
E-mail: Florian.Kronenberg@i-med.ac.at

Claudia Lamina
Division of Genetic Epidemiology
Department of Medical Genetics, Molecular and Clinical Pharmacology
Medical University of Innsbruck
Schöpfstr. 41, A-6020 Innsbruck, AUSTRIA
Phone: (+43) 512 9003-70565
Fax: (+43) 512 9003-73560 or -73561
E-mail: Claudia.Lamina@i-med.ac.at

Content

Supplementary Material and Methods 4
Study design and description of cohorts 4
Measurement of Lp(a) concentrations and apo(a) isoforms 5
Statistical methods 5
Quality control and filtering of SNPs 5
Meta-analysis 5
Gene-based candidate gene analysis 5
Bioinformatic analysis 6
Supplementary Tables 7
Supplementary Figures 33
Supplementary References 39

Supplementary Material and Methods

Study design and description of cohorts

The **CoLaus study** is a single-center, prospective study which included 6,182 Caucasian subjects aged 35 to 75 years from the city of Lausanne in Switzerland (1). The major aims of the CoLaus study are the investigation of prevalence, and determinants of cardiovascular disease and cardiovascular risk factors in the Lausanne population. Full phenotype information as well as imputed genotypes are available for n=3,964 participants and over 7.6 M SNPs after filtering.

For the **NHLBI Family Heart Study (FamHS),** 1,200 families (≈6,000 individuals) were ascertained in 1992 , half randomly sampled, half selected because of an excess of coronary heart disease (CHD) or risk factor abnormalities (2). Study participants belonging to the largest pedigrees were invited for a second clinical exam (2002-04). GWA analysis was undertaken for 4,135 European participants leading to data including in total about 8.5 M SNPs after imputation and filtering. Both imputed genotype data as well as phenotype information is available for n=1,712 participants.

The **KORA F3** study, conducted in the years 2004/05, is a population-based sample from the general population living in the region of Augsburg, Southern Germany, which has evolved from the WHO MONICA study (Monitoring of Trends and Determinants of Cardiovascular Disease). Genome-wide data are available for all participants (n=3,076 with complete phenotype information) and about 9.7 M SNPs after imputation and filtering.

The **KORA F4** survey is an independent non-overlapping sample drawn from the same population in the years 2006/08. Genome-wide data are available for all participants in the KORA F4 study (n=2,926 with complete phenotype information) (3) and about 9.7 M SNPs after imputation and filtering. In KORA F4, two SNPs in the *APOE* gene (rs7412 and rs429358) were genotyped de novo in a multiplex approach using the SEQUENOM MassArray platform and iPLEX Gold chemistry.

The **Cardiovascular Risk in Young Finns Study (YFS)** is a prospective multicenter study from Finland initiated in 1980 (Baseline age 3–18 years) with several follow-ups over 30 years to investigate childhood risk factors for cardiometabolic outcomes (4). For 2,200 participants in the 2001 follow-up (ages 24-39 years), genome-wide data are available (5), leading to a total of more than 9 M imputed genotypes after filtering. Full phenotype information as well as imputed genotypes are available for n=2,103 participants.

The **SAPHIR** study (Salzburg Atherosclerosis Prevention Program in subjects at High Individual Risk) is an observational study conducted in the years 1999-2002 involving 1,770 unrelated subjects from a healthy working population. Study participants were recruited by health-screening programs in companies in and around the Austrian city of Salzburg (6). De-novo genotyping was performed for two SNPs in the *APOE* gene (rs7412 and rs429358) using the Agena MassARRAY4 system and iPlex

chemistry. Call rate was >99% for both. Full phenotype and genotype information for the two SNPs is available for n=1,422 participants.

Measurement of Lp(a) concentrations and apo(a) isoforms

All measurements of Lp(a) concentrations as well as the apo(a) isoforms were performed in the same laboratory (Division of Genetic Epidemiology, Innsbruck, Austria) for all participating studies. Details on the measurement technique are given in (7). We applied a double-antibody enzyme-linked immunosorbent assay using an affinity-purified polyclonal apo(a) antibody for coating and the horseradish peroxidase-conjugated monoclonal 1A2 for detection. Apo(a) phenotyping was performed by sodium dodecyl sulfate-agarose gel electrophoresis (SDS agarose) under reducing conditions as described in (8). Electrophoresis was followed by immunoblotting using the monoclonal antibody 1A2 for detection of apo(a) isoforms.

Statistical methods

Quality control and filtering of SNPs

Filtering of SNPs and quality control of the meta-analysis were performed centrally and standardized by the study group from Innsbruck using the program EasyQC (9). SNPs were only included in the analysis, when fulfilling the following criteria: imputation quality ≥0.4 (e.g. IMPUTE info), minor allele frequency ≥0.5% and a p-value of the HWE-test ≥1e-06. Additional analyses for quality control were applied on the already filtered result datasets, which included the P-Z-plot (9) and calculation of genomic inflation factor λ. The P-Z-plot compares the reported p-values from each study with the p-values calculated from Z-statistics derived from the reported beta coefficient and standard error.

Meta-analysis

For the meta-analysis of all GWA studies, the software METASOFT (10) was used for all imputed SNPs that met imputation and quality control criteria. SNPs were only included in the meta-analysis if they were available in two or more studies. Per default, the fixed effects inverse variance-weighted method was applied to all SNPs. In addition, the optimized random effects model (RE) as proposed by Han & Eskin (10) was additionally used for heterogeneous SNPs ($I^2 \geq 50$). In each model, the test statistics were corrected for genomic inflation in both the GWA analysis stage and meta-analysis stage.

Gene-based candidate gene analysis

The 21 candidate genes that were tested for association are the reported *LPA* receptors and possible *LPA* regulators *LDLR* (11), *CD36* (12, 13), *LGALS1* (14), *LRP1* (aka *APOER/*apoE receptor) (15), *LRP2*

(aka megalin/gp330) (16), *LRP8 (*aka *APOER2*; a member of the LDLR family targeted by *PCSK9)* (17), *SCARB1* (18), *VLDLR* (19), *FBLN5* (20), *IL6* (21), *NR1H4 (*aka *FXR)* (22)*, FGF19* (23), *FGFR4* (23), *TLR2* (12)*, TLR4* (13)*,TLR6* (12, 24)*,* the plasminogen receptor *ANXA2 (*which has been implicated as potential Lp(a) receptor and in turn inhibits *PCSK9*) (25), as well as *PCSK9* (26) and components of its regulatory machinery (*SREBF1*, *SREBF2, HNF1A* (27)).

Bioinformatic analysis

By using the proxy search option of SNiPA(17), we retrieved all SNPs in LD with our hits reported in Supplementary Table S4 (1000g ph3v5 data with lower r^2 limit = 0.8; 499 unique SNPs). This SNP list was annotated using the UCSC Table Browser(28) (hg38.snp147CodingDbSnp table in the “All SNPs (147)” data track) to retrieve all SNPs located in protein-coding regions.The dbSNP functional category was reported by the Table Browser was followed for assignment to a canonical splice site. Only annotations referring to valid RefSeq Transcripts (NM-IDs) were considered. For *PLG* only transcript variant 1 (NM_000301) was considered as it is the larger transcript and transcript variant 2 (NM_001168338) shares the same coding region, but is truncated. Any annotations referring to predicted transcripts (XM-IDs) were discarded. Polyphen and SIFT predictions on missense variants were retrieved using the VEP tool(29).

The same SNP set was annotated also with ENCODE(30) and ORegAnno(31) data. The SNP list was used as input for UCSC Table browser (data track “All SNPs (147)”) and intersected with the wgEncodeRegTfbsClusteredV3 table in the data track “Txn Factor ChIP” and with the “ORegAnno data track”. Any positive intersections were reviewed manually in the UCSC Genome Browser and documented in Supplementary Table 10.

GTex(32) was searched for reported eQTL SNPs for *LPA* (i.e. SNP being correlated with *LPA* expression).

Supplementary Tables

Table S1: Baseline characteristics of contributing studies.

	CoLaus (n=3,964)	FamHS (n=1,712)	KORA F3 (n=3,076)	KORA F4 (n=2,926)	YFS (n=2,103)	SAPHIR (n=1,454)
Age (in years)	58.5 ± 10.5 [50, 58, 67]	51.2 ± 13.9 [39, 52, 63]	57.3 ± 12.9 [46, 57, 67]	56.2 ± 13.3 [44, 56, 67]	31.7 ± 5.0 [27, 33, 36]	51 ± 6.0 [46, 52, 55]
Women, n (%)	2124 (53.6%)	927 (54.1%)	1577 (51.27%)	1509 (51.6%)	1155 (54.9%)	472 (32.5%)
Lp(a) (mg/dL)	24 ± 27 [5, 12, 32]	21.0 ± 24.0 [4.2, 10.2, 32.5]	21.9 ± 26.0 [4.9, 11.1,28.4]	21.8 ± 24.6 [5.2, 11.7, 30.6]	12.5 ± 15.5 [2.8, 6.3, 15.1]	24.1 ± 27.5 [5.4, 11.8, 36.8]
LDL cholesterol (mg/dL)	133 ± 36 [108, 131, 158]	124.3 ± 33.8 [100, 123, 145]	128.0 ± 32.6 [105, 126, 148]	136.1 ± 34.9 [111, 134, 158]	126.2 ± 32.4 [104.4, 123.7, 147.0]	145.1 ± 35.6 [120, 144, 168]
HDL cholesterol (mg/dL)	64 ± 18 [50, 62, 73]	49.6 ± 14.7 [39, 47, 58]	58.8 ± 17.1 [47, 56, 69]	55.9 ± 14.4 [45, 54, 65]	49.8 ± 12.2 [41.0, 49.1, 57.6]	59.2 ± 15.6 [48, 57, 68]
Total cholesterol (mg/dL)	220 ± 40 [193, 220, 247]	203.6 ± 38.7 [177, 202, 228]	218.3 ± 39.9 [191, 216, 243]	216.1 ± 39.7 [188, 214, 240]	199.0 ± 37.7 [96.7, 197.2, 220.4]	226.7 ± 38.8 [200, 225, 251]
Triglycerides (mg/dL)	120 ± 78 [71, 97, 142]	148.8 ± 102.7 [84, 124, 186]	164.7 ± 125.8 [88, 135, 200]	125 ± 89.2 [72, 105, 151]	118.4 ± 75.7 [70.9, 97.4, 141.7]	126.4 ± 90 [72, 101, 151]

Continuous variables are shown as mean +/- stdev and [25%,50%,75%]-Percentiles

n: Sample size of analysis dataset with available Lp(a)-values and genotypes

Table S2: Genotyping and imputation information of contributing studies at the discovery stage.

	CoLaus	FamHS	KORA F3	KORA F4	YFS
SNP-chip	Affymetrix GeneChip® Human Mapping 500K	Illumina Human 1M-Duo v3 / Illumina 610-Quadv1/ Illumina Human Hap550K	Illumina Omni 2.5/ Illumina Omni Express	Affymetrix Axiom	Illumina 670k
QC before imputation	callrate >90%*, p-value HWE > 10^{-7}	callrate >98%, p-value HWE > 10^{-6}, MAF>1%	callrate >98%, p-value HWE > 10^{-10}	callrate >98%, p-value HWE > 10^{-10}	callrate >95%, p-value HWE > 10^{-6}, MAF>1%
Program used for Imputation	minimac	MACH	IMPUTE	IMPUTE	IMPUTE
Reference panel for imputation	1000G phase1v3	1000G phase1v3	1000G phase1v3	1000G phase1v3	1000G phase1v3
Program used for GWA analysis	In-house Matlab code	PROBABEL	SNPTest	SNPTest	SNPTest 2.4.1
Genomic inflation factor λ	1.011	1.030	1.021	1.015	1.011

*median call-rate for all SNPs used for imputation: 0.9957

Table S3: Definition and frequency of *APOE* genotypes.

rs7412	rs429358	*APOE**	n (%) KORA F3	n (%) KORA F4	n (%) SAPHIR
TT	TT	*E2/E2*	17 (0.55)	20 (0.70)	8 (0.47)
TT	TC	does not occur			
TT	CC	does not occur			
CT	TT	*E2/E3*	402 (13.06)	392 (13.74)	215 (12.72)
CT	TC	*E2/E4*	77 (2.50)	47 (1.65)	45 (2.66)
CT	CC	does not occur			
CC	TT	*E3/E3*	1,884 (61.23)	1,745 (61.19)	1,062 (62.84)
CC	TC	*E3/E4*	640 (20.80)	589 (20.65)	338 (20.00)
CC	CC	*E4/E4*	57 (1.85)	59 (2.07)	22 (1.30)

*according to (33, 34)

Table S4: Characteristics of the independent SNPs based on the conditional analysis for the inverse-normal transformed Lp(a) values adjusted for age and sex (model 1) as well as on the inverse-normal transformed Lp(a) values adjusted on age, sex and the predominantly expressed apo(a) isoform (model 2). The table is sorted by gene region and rsID. Column 8 reports coding SNPs in linkage disequilibrium (r^2=0.8) with the marker SNP. Missense SNPs are written in bold. When the marker SNP is coding itself, the complete marker line is written in bold and the column reports only the amino acid exchange.
The columns 9-11 report the number of correlated SNPs (including the lead SNP) which are located in any of the defined functional elements, based on UCSC Genome Browser data on hg19. Column 9 and 10 report transcription factor binding sites (TFBS) detected by ChIP(35–37) and DNase hypersensitive sites found by the ENCODE project(30). The ORegAnno(31) (column 10) is a curated database about regulatory regions. TFBS: transcription factor binding sites according to ChIP-Seq experiments. Details are reported in Table S9.

SNP	chr:position	nearest gene	Minor / ref allele	MAF range of contributing studies	MAF 1000G ph1v3	Independently associated in model		Correlated non-synonymous SNPs (r^2>0.8)	Number of correlated SNPs (r^2>0.8), which overlap with		
						1	2		ENCODE TFBS ChIP Signal	ORegAnno Site	ENCODE DNase hypersensitive cluster
***LPA* gene region**											
rs1018234	6:160796058	*SLC22A3*	T/C	0.288-0.318	0.286	✓			3		5
rs112842440	6:160955713	*LPA*	T/G	0.014-0.018	0.016		✓		1	1	
rs116850263	6:161029670	*LPA*	C/T	0.007-0.038	0.009	✓					
rs117026595	6:161068235	*LPA*	T/A	0.013-0.015	0.024		✓				
rs117162385	6:161078184	*LPA*	A/G	0.005-0.024	0.007	✓					
rs118039278	6:160985526	*LPA*	A/G	0.032-0.079	0.074	✓					
rs12204009	6:160799246	*SLC22A3*	C/T	0.005-0.008	0.009	✓			1		
rs12207325	6:160903911	*SLC22A3*	A/G	0.007-0.013	0.005		✓				
rs12529361	6:161125631	*PLG*	C/T	0.169-0.223	0.217	✓			1		
rs12664092	6:160948136	*LPA*	C/A	0.029-0.036	0.028	✓	✓				
rs139699952	6:161221380	*PLG*	C/G	0.025-0.030	0.046	✓			1		
rs140570886	6:161013013	*LPA*	C/T	0.011-0.023	0.011		✓	rs3798220 (*LPA* Iso1891Met)	1	1	2
rs141463285	6:160838646	*SLC22A3*	A/T	0.005-0.007	0.007	✓	✓		1		
rs141550222	6:160992001	*LPA*	G/C	0.023-0.062	0.040	✓					
rs142126734	6:160942926	*LPA*	A/G	0.035-0.054	0.042	✓	✓				
rs143520616	6:160631170	*SLC22A2*	T/G	0.009-0.026	0.032	✓					
rs143665477	6:160867272	*SLC22A3*	C/T	0.007-0.013	0.008	✓					
rs145470851	6:161004351	*LPA*	A/G	0.006-0.014	0.009		✓				

… to be continued

Table S4: continued

SNP	chr:position	nearest gene	Minor / ref allele	MAF range of contributing studies	MAF 1000G ph1v3	Indepen-dently associated in model		Correlated non-synonymous SNPs (r^2>0.8)	Number of correlated SNPs (r^2>0.8), which overlap with		
						1	2		ENCODE TFBS ChIP Signal	ORegAn no Site	ENCODE DNase hypersensitive cluster
rs147010904	6:160625299	*SLC22A2*	T/C	0.006-0.010	0.004		✓				
rs147402310	6:161163530	*PLG*	G/T	0.019-0.043	0.012	✓			1		
rs147555597	6:160911596	*SLC22A3*	A/G	0.006-0.007	0.007		✓				
rs149302195	6:160803694	*SLC22A3*	T/C	0.006-0.018	0.005		✓				
rs150571318	6:161702138	*AGPAT4*	A/G	0.006-0.026	0.005	✓			1		
rs1510224	6:160894878	*SLC22A3*	C/T	0.013-0.021	0.009	✓		rs3798220 (*LPA* Iso1891Met)	1	1	1
rs151181359	6:160934525	*LPA*	C/A	0.010-0.011	0.005	✓		rs41264848 (*LPA* Asp1719Asp)	15	3	
rs182532458	6:160453352	*IGF2R*	A/G	0.005-0.025	0.006	✓	✓		2	1	
rs184278183	6:161177756	*PLG*	T/C	0.005-0.010	0.003	✓					
rs185934663	6:161067929	*LPA*	G/T	0.005-0.018	0.009	✓					
rs186696265	6:161111700	*PLG*	T/C	0.009-0.017	0.011	✓	✓				
rs187270959	6:160340256	*MAS1*	C/A	0.005-0.011	0.005	✓					
rs188974863	6:161074983	*LPA*	A/T	0.082-0.102	0.093		✓				
rs2140650	6:161508438	*MAP3K4*	G/A	0.230-0.255	0.217	✓					
rs2246531	6:160838648	*SLC22A3*	G/A	0.193-0.242	0.229	✓			3		
rs2457557	6:160785507	*SLC22A3*	T/C	0.207-0.251	0.230	✓			4		
rs2457574	6:160868701	*SLC22A3*	A/G	0.427-0.531	0.435	✓					
rs2489940	6:161283929	*PLG*	T/G	0.010-0.032	0.011	✓					
rs3798221	6:160998148	*LPA*	T/G	0.189-0.240	0.212		✓		1		
rs41259144	6:161022107	*LPA*	T/C	0.006-0.012	0.013	✓		*LPA* Arg990Gln			
rs41267807	6:160952816	*LPA*	C/T	0.015-0.023	0.017		✓	*LPA* Tyr2023Cys			
rs41267809	6:160953642	*LPA*	G/A	0.006-0.024	0.021		✓	*LPA* Leu1961Pro			
rs41269133	6:161087863	*LPA*	C/T	0.079-0.156	0.115	✓					
rs41272114	6:161006077	*LPA*	T/C	0.023-0.048	0.028	✓	✓	LPA K-IV-8 5‘ splice site			
rs4252109	6:161137663	*PLG*	G/T	0.257-0.320	0.276	✓		rs4757 (*PLG* Asn110Asn), rs13231 (*PLG* Gln361Gln), rs4252125 (*PLG* Asp472Asn)	9	2	
rs4252185	6:161123451	*PLG*	C/T	0.052-0.078	0.073	✓					
rs4252198	6:161173728	*PLG*	G/C	0.014-0.022	0.024	✓	✓				

… to be continued

Table S4: continued

SNP	chr:position	nearest gene	Minor / ref allele	MAF range of contributing studies	MAF 1000G ph1v3	Indepen-dently associated in model 1	2	Correlated non-synonymous SNPs (r^2>0.8)	Number of correlated SNPs (r^2>0.8), which overlap with ENCODE TFBS ChIP Signal	ORegAn no Site	ENCODE DNase hypersensitive cluster
rs520829	6:160767905	*SLC22A3*	G/T	0.490-0.511	0.481	✓		rs668871 (*SLC22A3* Arg120Arg)	14	7	
rs55730499	6:161005610	*LPA*	T/C	0.032-0.078	0.070		✓				
rs56393506	6:161089307	*LPA*	T/C	0.152-0.172	0.171	✓	✓		1		
rs59614420	6:161129013	*PLG*	A/G	0.252-0.331	0.279		✓	rs4757 (*PLG* Asn110Asn), rs13231 (*PLG* Gln361Gln), rs4252125 (*PLG* Asp472Asn)	9	2	
rs614754	6:160505199	*IGF2R*	C/G	0.007-0.018	0.016	✓		*IGF2R* Leu2017Leu			
rs62440901	6:160569068	*SLC22A1*	T/C	0.123-0.162	0.148	✓	✓				
rs62441903	6:160987060	*LPA*	G/A	0.005-0.017	0.017	✓					
rs6902316	6:160893167	*SLC22A3*	G/A	0.009-0.015	0.002	✓		rs41264848 (*LPA* Asp1719Asp)	13	3	
rs6903649	6:160991798	*LPA*	C/T	0.247-0.342	0.284	✓					
rs6938647	6:160986915	*LPA*	A/C	0.201-0.268	0.219	✓	✓				
rs73596816	6:161017363	*LPA*	A/G	0.011-0.026	0.021	✓					
rs75234242	6:161018985	*LPA*	A/G	0.031-0.038	0.037		✓		1		
rs75692336	6:161030231	*LPA*	A/C	0.100-0.133	0.142		✓	rs41272110(38, 39) (*LPA* Thr1399Pro)	1		
rs76000021	6:160985501	*LPA*	C/T	0.014-0.023	0.016	✓					
rs7769879	6:160865645	*SLC22A3*	C/G	0.323-0.370	0.358		✓	rs2292334 (*SLC22A3* Ala411Ala)	2		
rs7770628	6:161018174	*LPA*	C/T	0.424-0.498	0.465	✓					
rs78439586	6:160820922	*SLC22A3*	A/G	0.059-0.077	0.063	✓	✓				
rs79246098	6:161078894	*LPA*	C/T	0.005-0.011	0.011	✓					
rs9295143	6:161654115	*AGPAT4*	G/C	0.053-0.074	0.047		✓				
rs9365169	6:160966347	*LPA*	G/C	0.495-0.536	0.488		✓				
rs9457778	6:160353291	*MAS1*	T/C	0.186-0.336	0.219	✓			12	1	
rs9458010	6:161125419	*PLG*	C/T	0.432-0.466	0.443	✓			1		
***APOE* gene region**											
rs7412	19:45412079	*APOE*	T/C	0.050-0.091	0.066	✓	✓				

Table S5: GWAS-results of the five cohorts on inverse-normal transformed Lp(a) values, adjusted on age and sex. The beta effect and effect direction refer to the minor allele. The Table is sorted according to the p-value of the meta-analysis results (as in Table S6).

			Colaus study		FamHS study		KORAF3 study		KORAF4 study		YFS study	
SNP	Min_allele/ Ref_allele	Effect Direction*	β (se)	p-value	β (se)	p-value	β (se)	p-value	β (se)	p-value	β (se)	p-value
***LPA* gene region**												
rs118039278	A/G	+++++	1.21 (0.059)	1.68E-94	1 (0.071)	1.59E-45	1.22 (0.059)	3.02E-89	1.27 (0.053)	3.95E-118	1.44 (0.091)	1.35E-53
rs56393506	T/C	+++++	0.75 (0.039)	1.13E-80	0.7 (0.055)	2.92E-36	0.72 (0.037)	2.49E-78	0.76 (0.037)	2.19E-88	0.39 (0.044)	1.79E-18
rs186696265	T/C	+++++	2.61 (0.139)	4.26E-78	1.59 (0.142)	3.63E-29	1.97 (0.125)	1.08E-53	1.81 (0.125)	7.88E-46	2.18 (0.16)	1.30E-40
rs4252185	C/T	+++++	1.18 (0.07)	1.30E-63	0.82 (0.077)	1.39E-26	0.85 (0.06)	1.20E-44	0.9 (0.053)	2.92E-62	0.83 (0.076)	4.37E-27
rs1510224	C/T	+++++	1.58 (0.095)	5.58E-62	1.26 (0.124)	3.30E-24	1.08 (0.1)	8.41E-27	1.37 (0.109)	5.93E-35	1.47 (0.138)	1.23E-25
rs6938647	A/C	-----	-0.39 (0.029)	1.03E-41	-0.33 (0.047)	2.33E-12	-0.28 (0.032)	1.52E-18	-0.41 (0.033)	4.49E-34	-0.41 (0.041)	2.06E-23
rs4252109	G/T	-----	-0.3 (0.024)	4.60E-37	-0.31 (0.037)	1.25E-16	-0.28 (0.027)	1.86E-23	-0.27 (0.028)	2.61E-21	-0.28 (0.034)	2.62E-16
rs41272114	T/C	-----	-0.87 (0.092)	2.87E-21	-0.82 (0.112)	2.81E-13	-0.73 (0.083)	4.17E-18	-0.76 (0.092)	1.21E-16	-0.75 (0.071)	1.48E-25
rs4252198	G/C	+++++	1.25 (0.118)	2.44E-26	1.11 (0.137)	5.09E-16	1.18 (0.125)	6.04E-21	1.04 (0.134)	1.21E-14	1.14 (0.141)	1.31E-15
rs12529361	C/T	+++++	0.37 (0.034)	3.88E-27	0.29 (0.048)	1.80E-09	0.32 (0.034)	5.06E-21	0.2 (0.036)	2.71E-08	0.34 (0.045)	4.07E-14
rs2457574	A/G	-----	-0.16 (0.023)	6.47E-12	-0.17 (0.034)	4.71E-07	-0.14 (0.026)	4.77E-08	-0.15 (0.027)	6.18E-09	-0.46 (0.03)	5.22E-52
rs151181359	C/A	++?+?	1.14 (0.121)	3.75E-21	1.31 (0.208)	3.51E-10			1.5 (0.127)	1.70E-31		
rs41269133	C/T	-----	-0.33 (0.042)	8.24E-15	-0.44 (0.066)	3.67E-11	-0.35 (0.044)	6.13E-16	-0.26 (0.049)	1.24E-07	-0.33 (0.043)	2.39E-14
rs12204009	C/T	+++++	1.93 (0.218)	8.62E-19	1.28 (0.223)	1.01E-08	1.49 (0.208)	8.92E-13	1.19 (0.203)	5.23E-09	1.51 (0.227)	3.12E-11
rs142126734	A/G	+++++	0.43 (0.055)	3.05E-15	0.38 (0.076)	4.71E-07	0.33 (0.07)	2.39E-06	0.48 (0.061)	1.82E-15	0.6 (0.084)	1.43E-12
rs78439586	A/G	-----	-0.63 (0.062)	2.25E-24	-0.35 (0.088)	8.45E-05	-0.25 (0.057)	1.11E-05	-0.42 (0.055)	2.21E-14	-0.46 (0.074)	3.16E-10
rs6903649	C/T	-----	-0.21 (0.027)	1.87E-14	-0.14 (0.041)	9.09E-04	-0.17 (0.029)	1.02E-08	-0.23 (0.031)	3.25E-14	-0.27 (0.037)	8.00E-13
rs7770628	C/T	+++++	0.15 (0.024)	6.26E-10	0.19 (0.035)	1.03E-07	0.2 (0.025)	1.68E-14	0.21 (0.026)	4.41E-15	0.14 (0.031)	3.35E-06
rs141550222	G/C	-----	-0.73 (0.086)	1.15E-17	-0.74 (0.115)	1.08E-10	-0.56 (0.089)	3.79E-10	-0.56 (0.09)	6.20E-10	-0.33 (0.066)	5.08E-07
rs143665477	C/T	?+++?			1.43 (0.197)	3.81E-13	1.44 (0.152)	8.09E-21	1.36 (0.196)	5.89E-12		
rs184278183	T/C	+++++	1.4 (0.198)	1.59E-12	0.6 (0.191)	1.79E-03	0.66 (0.153)	1.50E-05	0.63 (0.176)	3.33E-04	1.47 (0.159)	7.18E-20
rs62441903	G/A	-----	-0.9 (0.153)	3.97E-09	-0.78 (0.217)	3.11E-04	-0.46 (0.133)	5.94E-04	-0.96 (0.107)	3.12E-19	-1.25 (0.292)	1.82E-05
rs2489940	T/G	-----	-0.96 (0.162)	3.01E-09	-0.72 (0.213)	6.85E-04	-0.53 (0.147)	3.26E-04	-0.75 (0.15)	5.06E-07	-0.77 (0.097)	2.21E-15
rs147402310	G/T	?----			-0.52 (0.146)	3.29E-04	-0.5 (0.104)	1.55E-06	-0.68 (0.123)	3.28E-08	-0.73 (0.083)	1.45E-18
rs520829	G/T	-----	-0.16 (0.023)	7.52E-12	-0.16 (0.034)	2.64E-06	-0.11 (0.026)	1.10E-05	-0.16 (0.026)	7.36E-10	-0.09 (0.031)	2.62E-03

… to be continued

Table S5: continued

SNP	Min_allele/ Ref_allele	Effect Direction*	Colaus study β (se)	Colaus study p-value	FamHS study β (se)	FamHS study p-value	KORAF3 study β (se)	KORAF3 study p-value	KORAF4 study β (se)	KORAF4 study p-value	YFS study β (se)	YFS study p-value
***LPA* gene region**												
rs6902316	G/A	++++?	0.69 (0.099)	3.81E-12	0.81 (0.173)	2.58E-06	0.87 (0.137)	2.51E-10	0.68 (0.148)	5.30E-06		
rs41259144	T/C	-?---	-0.94 (0.203)	3.73E-06			-0.79 (0.165)	2.02E-06	-0.79 (0.118)	2.35E-11	-1.08 (0.256)	2.57E-05
rs73596816	A/G	??+++					0.82 (0.136)	2.05E-09	0.49 (0.102)	1.59E-06	0.92 (0.177)	2.66E-07
rs185934663	G/T	?----			-1.04 (0.216)	1.33E-06	-0.58 (0.232)	1.30E-02	-1.29 (0.251)	3.26E-07	-0.68 (0.126)	8.45E-08
rs141463285	A/T	++?+?	1.05 (0.163)	8.96E-11	0.91 (0.195)	2.66E-06			0.7 (0.18)	1.06E-04		
rs116850263	C/T	??+++					0.76 (0.182)	3.44E-05	0.36 (0.195)	6.58E-02	0.57 (0.079)	6.39E-13
rs1018234	T/C	----?	-0.12 (0.025)	1.04E-06	-0.1 (0.038)	8.97E-03	-0.11 (0.028)	8.10E-05	-0.13 (0.028)	3.15E-06		
rs614754	C/G	?++++			0.86 (0.147)	5.09E-09	0.53 (0.163)	1.17E-03	0.47 (0.194)	1.44E-02	1.09 (0.231)	2.36E-06
rs62440901	T/C	+++++	0.15 (0.036)	1.87E-05	0.23 (0.046)	3.70E-07	0.09 (0.036)	9.15E-03	0.13 (0.036)	2.92E-04	0.08 (0.047)	7.50E-02
rs76000021	C/T	-----	-0.39 (0.118)	1.02E-03	-0.66 (0.137)	1.55E-06	-0.54 (0.118)	4.04E-06	-0.23 (0.121)	5.26E-02	-0.26 (0.135)	5.55E-02
rs9458010	C/T	+++++	0.07 (0.025)	4.41E-03	0.13 (0.038)	7.42E-04	0.12 (0.028)	1.25E-05	0.1 (0.029)	1.15E-03	0.07 (0.033)	4.59E-02
rs187270959	C/A	+++++	0.75 (0.198)	1.72E-04	0.4 (0.195)	3.91E-02	0.6 (0.162)	2.29E-04	0.59 (0.15)	8.70E-05	0.24 (0.209)	2.57E-01
rs139699952	C/G	?++++			0.4 (0.128)	1.87E-03	0.18 (0.109)	9.86E-02	0.49 (0.098)	5.60E-07	0.39 (0.112)	5.78E-04
rs150571318	A/G	???++							0.36 (0.211)	8.92E-02	0.65 (0.101)	1.04E-10
rs143520616	T/G	+++++	0.34 (0.098)	4.76E-04	0 (0.139)	9.95E-01	0.24 (0.095)	9.66E-03	0.33 (0.091)	3.69E-04	0.36 (0.185)	5.22E-02
rs12664092	C/A	+++++	0.19 (0.075)	1.22E-02	0.26 (0.121)	3.01E-02	0.15 (0.072)	3.74E-02	0.08 (0.074)	2.87E-01	0.43 (0.095)	4.73E-06
rs117162385	A/G	?--?+			-0.15 (0.295)	6.21E-01	-0.05 (0.236)	8.18E-01			0.58 (0.102)	1.62E-08
rs182532458	A/G	?--?+			-0.07 (0.241)	7.87E-01	-0.32 (0.181)	8.24E-02			0.64 (0.098)	7.74E-11
rs79246098	C/T	+++++	0.47 (0.19)	1.38E-02	0.14 (0.148)	3.55E-01	0.28 (0.135)	3.79E-02	0.18 (0.13)	1.67E-01	0.58 (0.212)	6.50E-03
rs2457557	T/C	---++	-0.12 (0.028)	2.76E-05	-0.07 (0.042)	1.10E-01	-0.03 (0.029)	3.00E-01	0 (0.031)	9.63E-01	0.1 (0.038)	9.78E-03
rs9457778	T/C	---+-	-0.05 (0.031)	8.92E-02	-0.02 (0.045)	6.60E-01	-0.02 (0.032)	5.74E-01	0.03 (0.034)	3.44E-01	-0.18 (0.033)	6.23E-08
rs2140650	G/A	++-++	0.01 (0.027)	6.72E-01	0.04 (0.04)	3.42E-01	-0.03 (0.03)	3.45E-01	0.01 (0.031)	6.95E-01	0.28 (0.036)	3.51E-14
rs2246531	G/A	----+	-0.08 (0.028)	2.25E-03	-0.06 (0.042)	1.48E-01	-0.03 (0.03)	3.15E-01	-0.01 (0.031)	8.23E-01	0.3 (0.039)	2.50E-14
***APOE* gene region**												
rs7412	T/C	?----	-0.62 (0.223)	5.15E-03	-0.18 (0.072)	1.25E-02	-0.22 (0.047)	1.69E-06	-0.12 (0.056)	2.89E-02	-0.22 (0.073)	2.55E-03

All p-values are based on the inverse normal transformed Lp(a) values. All effect estimates refer to the minor allele derived from 1000G, phase1v3 (see Supplementary Table 3);
*Order of included GWA studies: CoLaus, FamHS, KORA F3, KORA F4, YFS. +: positive effect from minor allele on inverse-normal transformed Lp(a) in that specific study, -: inverse effect from minor allele on inverse-normal transformed Lp(a) in that specific study;?: SNP not available in that study

Table S6: Results of the meta-analysis on the five cohorts on inverse-normal transformed Lp(a) values as well as Lp(a) on the original scale, adjusted on age and sex (model 1). The beta effect and effect direction refer to the minor allele. The Table is sorted by p-value.

			Single SNP analysis, fixed effects model			Single SNP analysis, heterogeneity and random effects model				Joint SNP analysis, fixed effects model, adjusted for all 49 independent SNPs		
SNP	Min_allele/ Ref_allele	Effect Direction*	β (se) INT scale	β (se) original scale	p-value	I^2	β_{RE} (se_{RE}) INT scale	β_{RE} (se_{RE}) original scale	p-value$_{RE}$	β_{Joint} (se_{Joint}) INT scale	β_{Joint} (se_{Joint}) original scale	p-value$_{Joint}$
***LPA* gene region**												
rs118039278	A/G	+++++	1.22 (0.029)	32.43 (0.657)	1.36e-396	75.2	1.22 (0.059)	32.15 (2.366)	0.00E+00	0.52(0.044)	16.92(1.051)	2.34E-32
rs56393506	T/C	+++++	0.68 (0.019)	13.6 (0.419)	7.76E-285	91.8	0.66 (0.066)	15.42 (3.026)	7.02E-291	0.17(0.027)	2.85(0.593)	1.88E-10
rs186696265	T/C	+++++	2.02 (0.062)	64.74 (1.341)	4.15E-231	86.4	2.03 (0.17)	67.48 (8.602)	1.47E-233	1.53(0.079)	47.6(1.792)	1.28E-84
rs4252185	C/T	+++++	0.92 (0.03)	23.93 (0.656)	5.31E-211	77.3	0.92 (0.063)	25.65 (3.644)	2.21E-211	0.29(0.039)	7.6(0.904)	4.35E-14
rs1510224	C/T	+++++	1.35 (0.05)	45.84 (1.115)	4.27E-160	71.7	1.35 (0.096)	45.98 (4.627)	2.95E-160	0.68(0.062)	26.38(1.416)	3.79E-28
rs6938647	A/C	-----	-0.37 (0.016)	-5.47 (0.362)	7.48E-119	61.6	-0.36 (0.026)	-5.57 (0.512)	1.66E-118	-0.25(0.026)	4.35(0.58)	6.21E-22
rs4252109	G/T	-----	-0.29 (0.013)	-4.91 (0.303)	4.86E-105	0				-0.17(0.017)	-3.11(0.378)	3.15E-25
rs41272114	T/C	-----	-0.78 (0.04)	-8.63 (0.821)	4.74E-86	0				-0.48(0.043)	-2.43(0.881)	7.98E-29
rs4252198	G/C	+++++	1.15 (0.059)	27.97 (1.316)	2.72E-84	0				0.76(0.062)	18.07(1.394)	8.32E-34
rs12529361	C/T	+++++	0.3 (0.017)	4.97 (0.394)	5.57E-69	68.8	0.3 (0.031)	4.95 (0.922)	4.29E-69	0.27(0.02)	4.8(0.445)	3.77E-42
rs2457574	A/G	-----	-0.21 (0.012)	-4.75 (0.277)	2.77E-63	95.5	-0.22 (0.058)	-4.5 (0.575)	1.76E-77	-0.2(0.02)	-5.08(0.422)	1.80E-25
rs151181359	C/A	++?+?	1.31 (0.082)	39.56 (2.035)	1.55E-57	51.1	1.32 (0.123)	38.58 (5.875)	4.47E-57	0.56(0.09)	10.41(2.257)	3.66E-10
rs41269133	C/T	-----	-0.33 (0.021)	-5.72 (0.457)	5.42E-55	15.4				-0.2(0.028)	-2.59(0.591)	1.79E-13
rs12204009	C/T	+++++	1.47 (0.098)	32.86 (2.319)	2.80E-51	42.7				0.66(0.102)	11.49(2.408)	7.00E-11
rs142126734	A/G	+++++	0.44 (0.03)	6.58 (0.699)	8.52E-48	43				0.31(0.035)	4.1(0.798)	7.42E-19
rs78439586	A/G	-----	-0.42 (0.029)	-6.68 (0.661)	1.28E-47	80.8	-0.42 (0.068)	-7.04 (1.214)	4.83E-49	-0.27(0.032)	-3.99(0.724)	1.70E-17
rs6903649	C/T	-----	-0.2 (0.014)	-2.6 (0.33)	3.91E-45	49.5				-0.18(0.028)	1.55(0.634)	8.27E-11
rs7770628	C/T	+++++	0.18 (0.012)	4.73 (0.279)	4.80E-45	9.5				0.31(0.021)	-7.28(0.482)	8.08E-47
rs141550222	G/C	-----	-0.54 (0.039)	-6.71 (0.781)	6.73E-44	77.7	-0.57 (0.084)	-9.29 (2.253)	6.10E-45	-0.32(0.044)	-1.73(0.891)	5.69E-13
rs143665477	C/T	?+++?	1.41 (0.105)	39.71 (2.611)	2.63E-41	0				0.68(0.11)	20.65(2.757)	8.28E-10
rs184278183	T/C	+++++	0.95 (0.079)	26.25 (1.663)	9.36E-34	84.1	0.95 (0.198)	25.27 (4.554)	7.03E-36	0.66(0.082)	21.34(1.743)	8.01E-16
rs62441903	G/A	-----	-0.82 (0.069)	-14.36 (1.657)	4.86E-33	64.5	-0.83 (0.125)	-14.36 (1.657)	4.38E-33	-0.69(0.07)	-10.36(1.688)	6.83E-23
rs2489940	T/G	-----	-0.75 (0.063)	-8.69 (1.231)	1.67E-32	0				-0.44(0.065)	-5.11(1.268)	1.07E-11
rs147402310	G/T	?----	-0.63 (0.054)	-7.19 (1.053)	8.97E-32	19.5				-0.51(0.055)	-5.58(1.064)	1.21E-20
rs520829	G/T	-----	-0.14 (0.012)	-3.58 (0.28)	2.65E-29	17.7				-0.16(0.022)	-2.06(0.452)	2.34E-12

… to be continued

Table S6: continued

			Single SNP analysis, fixed effects model			Single SNP analysis, heterogeneity and random effects model				Joint SNP analysis, fixed effects model, adjusted for all 49 independent SNPs		
SNP	Min_allele/ Ref_allele	Effect Direction*	β (se) INT scale	β (se) original scale	p-value	I^2	β_{RE} (se_{RE}) INT scale	β_{RE} (se_{RE}) original scale	p-value$_{RE}$	β_{Joint} (se_{Joint}) INT scale	β_{Joint} (se_{Joint}) original scale	p-value$_{Joint}$
***LPA* gene region**												
rs6902316	G/A	++++?	0.74 (0.066)	19.15 (1.684)	4.30E-29	0				0.46(0.071)	11.93(1.813)	8.45E-11
rs41259144	T/C	-?---	-0.85 (0.084)	-14.27 (1.936)	4.45E-24	0				-0.6(0.085)	-7.52(1.963)	2.01E-12
rs73596816	A/G	??+++	0.66 (0.075)	11.2 (1.664)	1.53E-18	66.8	0.72 (0.138)	11.57 (2.267)	1.80E-18	0.58(0.078)	10.33(1.705)	9.61E-14
rs185934663	G/T	?----	-0.81 (0.093)	-6.83 (1.709)	4.58E-18	54.8	-0.86 (0.152)	-8.56 (2.725)	8.04E-18	-0.64(0.095)	-4.94(1.733)	1.44E-11
rs141463285	A/T	++?+?	0.9 (0.104)	24.12 (2.622)	6.28E-18	3.2				0.65(0.108)	20.82(2.705)	1.87E-09
rs116850263	C/T	??+++	0.57 (0.069)	4.89 (1.169)	1.01E-16	6.1				0.42(0.07)	2.9(1.182)	1.42E-09
rs1018234	T/C	----?	-0.12 (0.015)	-2.49 (0.372)	6.30E-16	0				0.3(0.029)	4.63(0.608)	3.20E-24
rs614754	C/G	?++++	0.72 (0.09)	16.12 (2.003)	1.43E-15	52.7	0.72 (0.133)		2.39E-15	0.58(0.091)	-12.1(2.026)	2.35E-10
rs62440901	T/C	+++++	0.14 (0.018)	2.05 (0.405)	3.11E-14	42.9				0.12(0.018)	2.62(0.415)	5.39E-11
rs76000021	C/T	-----	-0.41 (0.057)	-7.54 (1.266)	4.70E-13	47.9				-0.41(0.058)	-5.84(1.291)	2.28E-12
rs9458010	C/T	+++++	0.09 (0.014)	2.84 (0.302)	4.11E-12	0				0.14(0.019)	3.01(0.428)	6.02E-13
rs187270959	C/A	+++++	0.53 (0.081)	12.38 (1.828)	4.63E-11	0				0.57(0.082)	13.15(1.845)	3.81E-12
rs139699952	C/G	?++++	0.37 (0.056)	6.79 (1.187)	4.70E-11	33.1				0.33(0.057)	6.92(1.198)	5.79E-09
rs150571318	A/G	???++	0.6 (0.092)	6.55 (1.514)	6.90E-11	35				0.54(0.093)	5.82(1.522)	6.59E-09
rs143520616	T/G	+++++	0.27 (0.05)	2.39 (1.194)	5.01E-08	14.9				0.31(0.05)	3.82(1.205)	9.63E-10
rs12664092	C/A	+++++	0.2 (0.038)	5.4 (0.846)	1.92E-07	57.1	0.21 (0.059)	5.42 (1.599)	1.81E-07	0.5(0.043)	11.41(0.959)	3.67E-31
rs117162385	A/G	?--?+	0.43 (0.091)	2.78 (1.522)	2.71E-06	79.5	0.18 (0.268)	-1.24 (4.438)	6.50E-07	0.53(0.091)	4.66(1.525)	6.60E-09
rs182532458	A/G	?--?+	0.37 (0.082)	4.36 (1.425)	5.75E-06	91.8	0.1 (0.344)	-0.87 (4.864)	2.57E-09	0.7(0.084)	8.06(1.438)	7.41E-17
rs79246098	C/T	+++++	0.28 (0.07)	0.24 (1.627)	7.53E-05	7.4				0.4(0.072)	5.15(1.661)	2.18E-08
rs2457557	T/C	---++	-0.05 (0.016)	-2.39 (0.403)	5.60E-04	65.6	-0.05 (0.028)	-2.41 (0.616)	2.76E-04	-0.25(0.033)	2.72(0.682)	1.17E-13
rs9457778	T/C	---+-	-0.05 (0.015)	-0.05 (0.336)	8.75E-04	81.8	-0.05 (0.037)	0.31 (0.676)	7.28E-06	-0.11(0.016)	-2.17(0.344)	1.16E-12
rs2140650	G/A	++-++	0.05 (0.015)	0.94 (0.328)	1.10E-03	91.7	0.06 (0.051)	0.54 (0.86)	1.62E-10	0.09(0.015)	-1.97(0.336)	5.04E-10
rs2246531	G/A	----+	0 (0.015)	-1.04 (0.339)	8.48E-01	94.3	0.02 (0.063)	-1.47 (0.997)	2.17E-12	0.31(0.034)	-2.2(0.653)	1.67E-19
***APOE* gene region**												
rs7412	T/C	?----	-0.19 (0.030)	-3.34 (0.668)	3.47e-10	0						

All p-values are based on the inverse normal transformed Lp(a) values. All effect estimates refer to the minor allele derived from 1000G, phase1v3 (see Supplementary Table 3);
*Order of included GWA studies: CoLaus, FamHS, KORA F3, KORA F4, YFS. +: positive effect from minor allele on inverse-normal transformed Lp(a) in that specific study, -: inverse effect from minor allele on inverse-normal transformed Lp(a) in that specific study;?: SNP not available in that study

Table S7: Results of the meta-analysis on the five cohorts on inverse-normal transformed Lp(a) values adjusted on age, sex and the predominantly expressed apo(a) isoform (model 2). The beta effect and effect direction refer to the minor allele. The Table is sorted according to the p-value of the meta-analysis results (as in Table S8).

			Colaus study		FamHS study		KORAF3 study		KORAF4 study		YFS study	
SNP	Min_allele/ Ref_allele	Effect Direction*	β (se)	p-value	β (se)	p-value	β (se)	p-value	β (se)	p-value	β (se)	p-value
***LPA* gene region**												
rs75692336	A/C	-----	-0.65 (0.036)	3.81E-74	-0.19 (0.044)	1.18E-05	-0.61 (0.036)	2.03E-61	-0.64 (0.038)	6.97E-63	-0.61 (0.05)	1.41E-33
rs140570886	C/T	+++++	2.01 (0.108)	5.38E-77	0.96 (0.103)	9.75E-21	1.77 (0.113)	5.91E-53	1.58 (0.114)	5.88E-42	1.94 (0.151)	2.03E-36
rs186696265	T/C	+++++	2.59 (0.139)	4.12E-78	1.02 (0.116)	2.84E-18	1.94 (0.125)	5.58E-52	1.76 (0.126)	4.21E-43	2.14 (0.16)	2.94E-39
rs7769879	C/G	+++++	0.36 (0.024)	1.51E-52	0.23 (0.029)	2.37E-15	0.35 (0.026)	6.73E-40	0.28 (0.027)	1.71E-23	0.49 (0.032)	1.65E-51
rs55730499	T/C	+++++	0.78 (0.064)	1.21E-34	0.47 (0.059)	2.23E-15	0.92 (0.062)	5.16E-49	0.82 (0.054)	1.17E-49	1.07 (0.094)	4.63E-29
rs188974863	A/T	-----	-0.76 (0.052)	1.03E-48	-0.19 (0.059)	9.29E-04	-0.51 (0.049)	2.35E-25	-0.53 (0.052)	1.25E-24	-0.54 (0.055)	7.12E-23
rs56393506	T/C	+++++	0.45 (0.04)	1.49E-29	0.31 (0.045)	2.33E-12	0.47 (0.039)	2.96E-33	0.46 (0.038)	9.46E-33	0.26 (0.045)	7.35E-09
rs75234242	A/G	-----	-1.05 (0.08)	3.63E-39	-0.42 (0.102)	3.37E-05	-0.69 (0.078)	7.37E-19	-0.77 (0.079)	6.08E-22	-0.91 (0.088)	1.86E-24
rs6938647	A/C	-----	-0.28 (0.029)	1.25E-21	-0.35 (0.038)	8.14E-20	-0.25 (0.032)	1.23E-14	-0.32 (0.033)	1.50E-21	-0.37 (0.041)	7.36E-19
rs59614420	A/G	-----	-0.25 (0.024)	6.27E-24	-0.24 (0.031)	1.65E-14	-0.23 (0.028)	4.43E-17	-0.22 (0.028)	2.01E-14	-0.27 (0.035)	2.45E-14
rs142126734	A/G	+++++	0.52 (0.054)	4.12E-22	0.44 (0.062)	9.96E-13	0.31 (0.07)	9.45E-06	0.53 (0.061)	4.25E-18	0.43 (0.085)	3.51E-07
rs4252198	G/C	+++++	0.81 (0.118)	7.29E-12	0.53 (0.114)	4.14E-06	0.87 (0.126)	6.03E-12	0.61 (0.135)	6.87E-06	0.9 (0.142)	2.82E-10
rs12207325	A/G	-----	-1.14 (0.163)	3.06E-12	-0.48 (0.158)	2.28E-03	-1.09 (0.128)	2.23E-17	-0.64 (0.173)	2.38E-04	-1.46 (0.231)	2.92E-10
rs145470851	A/G	-----	-1.22 (0.136)	3.08E-19	-0.26 (0.175)	1.45E-01	-0.75 (0.151)	6.76E-07	-0.86 (0.136)	2.93E-10	-1.15 (0.248)	3.93E-06
rs9365169	G/C	-----	-0.16 (0.023)	1.05E-12	-0.06 (0.028)	3.31E-02	-0.21 (0.025)	1.82E-16	-0.15 (0.026)	1.10E-08	-0.06 (0.031)	6.09E-02
rs78439586	A/G	-----	-0.47 (0.062)	5.28E-14	-0.33 (0.072)	4.55E-06	-0.19 (0.058)	8.43E-04	-0.3 (0.055)	9.66E-08	-0.34 (0.075)	4.59E-06
rs117026595	T/A	??---					-0.96 (0.128)	9.80E-14	-0.71 (0.135)	1.65E-07	-1.08 (0.163)	5.65E-11
rs41272114	T/C	-----	-0.49 (0.092)	1.02E-07	-0.58 (0.094)	9.98E-10	-0.38 (0.088)	1.69E-05	-0.25 (0.097)	9.94E-03	-0.36 (0.078)	3.83E-06
rs112842440	T/G	+++++	0.67 (0.122)	4.44E-08	0.57 (0.111)	3.09E-07	0.45 (0.109)	3.99E-05	0.51 (0.104)	8.59E-07	0.37 (0.126)	3.02E-03
rs12664092	C/A	+++++	0.44 (0.075)	4.13E-09	0.32 (0.098)	1.28E-03	0.35 (0.072)	1.01E-06	0.25 (0.074)	6.49E-04	0.48 (0.094)	5.16E-07
rs147010904	T/C	++++?	0.9 (0.149)	1.28E-09	0.46 (0.152)	2.51E-03	0.8 (0.161)	7.90E-07	0.78 (0.181)	1.88E-05		
rs149302195	T/C	-?---	-0.82 (0.182)	6.15E-06			-0.68 (0.2)	7.47E-04	-0.39 (0.148)	8.14E-03	-0.79 (0.128)	6.22E-10
rs141463285	A/T	++?+?	1.06 (0.162)	6.12E-11	0.61 (0.157)	9.20E-05			0.58 (0.181)	1.40E-03		
rs147555597	A/G	+++??	0.79 (0.168)	2.47E-06	0.8 (0.182)	1.08E-05	0.7 (0.161)	1.43E-05				
rs41267809	G/A	-----	-0.18 (0.122)	1.40E-01	-0.4 (0.135)	3.06E-03	-0.21 (0.11)	5.90E-02	-0.54 (0.085)	2.51E-10	-0.1 (0.271)	7.21E-01
rs9295143	G/C	+++++	0.18 (0.075)	1.43E-02	0.24 (0.072)	6.74E-04	0.15 (0.067)	2.09E-02	0.12 (0.061)	5.49E-02	0.2 (0.064)	2.24E-03
rs41267807	C/T	+----	0.03 (0.113)	8.21E-01	-0.39 (0.109)	3.39E-04	-0.26 (0.119)	2.86E-02	-0.19 (0.119)	1.14E-01	-0.78 (0.134)	7.30E-09
rs182532458	A/G	?--?+			-0.03 (0.195)	8.77E-01	-0.05 (0.181)	7.97E-01			0.46 (0.099)	2.92E-06

… to be continued

Table S7: continued

SNP	Min_allele/ Ref_allele	Effect Direction*	Colaus study β (se)	Colaus study p-value	FamHS study β (se)	FamHS study p-value	KORAF3 study β (se)	KORAF3 study p-value	KORAF4 study β (se)	KORAF4 study p-value	YFS study β (se)	YFS study p-value
***LPA* gene region**												
rs62440901	T/C	++-+-	0.08 (0.036)	2.19E-02	0.15 (0.037)	5.60E-05	-0.01 (0.036)	7.12E-01	0.03 (0.036)	4.18E-01	-0.03 (0.048)	4.98E-01
rs3798221	T/G	--+-+	-0.04 (0.029)	1.55E-01	-0.1 (0.035)	5.92E-03	0.01 (0.033)	8.25E-01	-0.04 (0.033)	1.79E-01	0.05 (0.036)	1.45E-01
***APOE* gene region**												
rs7412	T/C	?----			-0.12 (0.058)	4.72E-02	-0.25 (0.047)	6.09E-08	-0.08 (0.056)	1.45E-01	-0.19 (0.074)	9.68E-03

All p-values are based on the inverse-normal transformed Lp(a) values. All effect estimates refer to the minor allele derived from 1000G, phase1v3 (see Supplementary Table 3);
*Order of included GWA studies: CoLaus, FamHS, KORA F3, KORA F4, YFS. +: positive effect from minor allele on inverse-normal transformed Lp(a) in that specific study, -: inverse effect from minor allele on inverse-normal transformed Lp(a) in that specific study;?: SNP not available in that study

Table S8: Results of the meta-analysis on the five cohorts on inverse-normal transformed Lp(a) values adjusted on age, sex and the predominantly expressed apo(a) isoform (model 2). The beta effect and effect direction refer to the minor allele. The Table is sorted by p-value.

			**Single SNP analysis, fixed effects model**			**Single SNP analysis, heterogeneity and random effects model**				**Joint SNP analysis, fixed effects model, adjusted for all 31 independent SNPs**		
**SNP**	**Min_allele/ Ref_allele**	**Effect Direction***	**β (se) INT scale**	**β (se) original scale**	**p-value**	**I^2**	**β_{RE} (se_{RE}) INT scale**	**β_{RE} (se_{RE}) original scale**	**p-value$_{RE}$**	**β_{Joint} (se_{Joint}) INT scale**	**β_{Joint} (se_{Joint}) original scale**	**p-value$_{Joint}$**
***LPA* gene region**												
rs75692336	A/C	-----	-0.56 (0.018)	-9.67 (0.358)	2.90E-216	95.2	-0.54 (0.082)	-9.89 (1.512)	4.30E-229	-0.46(0.024)	-7.38(0.469)	4.91E-85
rs140570886	C/T	+++++	1.61 (0.052)	43.43 (1.008)	1.72E-210	93.2	1.65 (0.2)	43.85 (4.736)	1.16E-218	0.83(0.076)	23.78(1.519)	7.49E-28
rs186696265	T/C	+++++	1.82 (0.059)	49.06 (1.129)	2.08E-207	95.1	1.89 (0.269)	51.13 (6.921)	3.09E-220	0.85(0.088)	24.75(1.755)	4.30E-22
rs7769879	C/G	+++++	0.34 (0.012)	6.74 (0.238)	4.43E-168	91	0.34 (0.041)	6.79 (0.451)	2.80E-173	0.24(0.019)	4.69(0.373)	1.05E-35
rs55730499	T/C	+++++	0.78 (0.029)	16.77 (0.584)	3.97E-163	90.4	0.81 (0.094)	16.84 (1.311)	7.57E-168	0.39(0.036)	10.18(0.722)	3.24E-27
rs188974863	A/T	-----	-0.53 (0.024)	-9.13 (0.452)	1.92E-107	92.3	-0.51 (0.086)	-10.01 (1.617)	3.37E-114	-0.29(0.026)	-5.17(0.496)	2.25E-28
rs56393506	T/C	+++++	0.4 (0.019)	7.07 (0.356)	2.81E-105	80.3	0.4 (0.042)	7.92 (1.578)	2.87E-106	0.17(0.024)	3.12(0.445)	7.77E-13
rs75234242	A/G	-----	-0.79 (0.038)	-13.59 (0.721)	3.78E-97	85.1	-0.77 (0.099)	-14.52 (1.909)	3.40E-99	-0.39(0.042)	-6.49(0.802)	1.36E-20
rs6938647	A/C	-----	-0.3 (0.015)	-5.48 (0.3)	2.36E-87	45.5				-0.27(0.019)	4.12(0.377)	5.75E-43
rs59614420	A/G	-----	-0.24 (0.013)	-4.48 (0.253)	1.39E-76	0				-0.13(0.015)	-2.11(0.289)	5.85E-18
rs142126734	A/G	+++++	0.46 (0.029)	7.77 (0.577)	2.70E-57	45.5				0.35(0.031)	6.09(0.615)	2.42E-29
rs4252198	G/C	+++++	0.73 (0.057)	14.9 (1.105)	3.69E-38	42.6				0.5(0.058)	10.09(1.138)	9.43E-18
rs12207325	A/G	-----	-0.93 (0.073)	-14.8 (1.48)	2.50E-37	78.9	-0.95 (0.163)	-14.67 (1.812)	2.87E-38	-0.71(0.076)	-10.49(1.539)	1.53E-20
rs145470851	A/G	-----	-0.86 (0.071)	-15.97 (1.449)	8.83E-34	80.2	-0.84 (0.164)	-15.42 (2.957)	5.17E-35	-0.5(0.075)	-9.76(1.514)	2.34E-11
rs9365169	G/C	-----	-0.14 (0.012)	-2.05 (0.231)	4.23E-31	82.7	-0.13 (0.029)	-2.27 (0.594)	7.89E-33	0.14(0.019)	-2.91(0.36)	2.53E-13
rs78439586	A/G	-----	-0.32 (0.028)	-5.91 (0.551)	3.30E-29	62.7	-0.32 (0.047)	-6.18 (1.007)	3.29E-29	-0.17(0.031)	-3.34(0.589)	2.32E-08
rs117026595	T/A	??---	-0.9 (0.082)	-13.81 (1.45)	2.61E-28	39				-0.68(0.084)	-10.07(1.48)	2.61E-16
rs41272114	T/C	-----	-0.41 (0.04)	-5.73 (0.723)	2.97E-24	41.8				-0.38(0.042)	-4.7(0.759)	7.95E-20
rs112842440	T/G	+++++	0.51 (0.051)	6.85 (0.984)	1.06E-23	0				0.39(0.053)	5.4(1.01)	2.04E-13
rs12664092	C/A	+++++	0.36 (0.036)	7.17 (0.701)	1.72E-23	17.6				0.34(0.039)	6.35(0.739)	1.85E-18
rs147010904	T/C	++++?	0.73 (0.081)	18.38 (1.783)	9.25E-20	35.5				0.53(0.089)	13.04(1.968)	2.93E-09
rs149302195	T/C	-?---	-0.67 (0.079)	-10.14 (1.312)	2.32E-17	41.2				-0.48(0.08)	-7.71(1.328)	2.89E-09
rs141463285	A/T	++?+?	0.76 (0.097)	17.96 (2.151)	3.27E-15	61.6	0.75 (0.156)	17.81 (4.066)	4.52E-15	0.6(0.098)	15.42(2.178)	9.42E-10
rs147555597	A/G	+++??	0.76 (0.099)	21.1 (2.227)	1.63E-14	0				0.63(0.109)	17(2.453)	7.04E-09
rs41267809	G/A	-----	-0.36 (0.053)	-6.76 (1.112)	2.95E-11	57.8	-0.32 (0.088)	-6.37 (1.38)	1.97E-11	-0.33(0.055)	-5.89(1.145)	3.20E-09
rs9295143	G/C	+++++	0.17 (0.03)	3.01 (0.558)	8.23E-09	0				0.18(0.031)	-3.09(0.563)	3.74E-09
rs41267807	C/T	+----	-0.29 (0.053)	-5.32 (1.035)	3.25E-08	82.3	-0.31 (0.127)	-4.93 (1.548)	4.02E-10	-0.34(0.055)	-6.43(1.061)	5.10E-10
rs182532458	A/G	?--?+	0.29 (0.08)	3.75 (1.188)	3.04E-04	77.8	0.16 (0.194)	0.22 (3.563)	8.11E-05	0.51(0.081)	6.46(1.199)	3.11E-10
rs62440901	T/C	++-+-	0.05 (0.017)	0.91 (0.337)	4.65E-03	71.5	0.05 (0.032)	1.05 (0.55)	9.10E-04	0.14(0.018)	2.6(0.348)	3.92E-16
rs3798221	T/G	--+-+	-0.04 (0.016)	-1.25 (0.357)	1.06E-02	34.1				-0.23(0.02)	-3.53(0.418)	1.09E-31

… to be continued

Table S8: continued

SNP	Min_allele/ Ref_allele	Effect Direction*	Single SNP analysis, fixed effects model: β (se) INT scale	β (se) original scale	p-value	Single SNP analysis, heterogeneity and random effects model: I²	β_{RE} (se_{RE}) INT scale	β_{RE} (se_{RE}) original scale	p-value$_{RE}$	Joint SNP analysis, fixed effects model, adjusted for all 31 independent SNPs: β_{Joint} (se_{Joint}) INT scale	β_{Joint} (se_{Joint}) original scale	p-value$_{Joint}$
***APOE* gene region**												
rs7412	T/C	?----	-0.17 (0.029)	-2.78 (0.555)	3.48e-09	53.9	-0.16 (0.043)	-2.68 (0.908)	3.32e-09			

All p-values are based on the inverse-normal transformed Lp(a) values. All effect estimates refer to the minor allele derived from 1000G, phase1v3 (see Supplementary Table 3);
*Order of included GWA studies: CoLaus, FamHS, KORA F3, KORA F4, YFS. +: positive effect from minor allele on inverse-normal transformed Lp(a) in that specific study, -: inverse effect from minor allele on inverse-normal transformed Lp(a) in that specific study;?: SNP not available in that study

Table S9: This table reports detailed data for functional elements of table 4. All SNPs in LD (r^2>0.8) with each lead SNP of our association study, which is located at any functional element, is reported (LD data according to SNiPA(17)). For each SNP location transcription factor binding sites (TFBS) detected by ChIP by the ENCODE project, curated functional elements from the ORegAnno(31) database and DNase hypersensitive sites according to ENCODE data are reported. Several SNPs are located in clusters of TFBS, which is visible by annotation of dozens of TFNBS to a SNP. Some of these are validated by the ORegAnno(31) database information and by colocation with DNase hypersensitive sites.

Proxy SNP (r^2>0.8)	Lead SNP (model 1 or 2)	R2	d'	MAF	Located in TFBS element	Located in ORegAnno element	Located in DNase hypersensitive cluster
rs2661845	rs1018234	0.84	0.93	0.2813			yes
rs402219	rs1018234	0.93	0.98	0.2843	CTCF, RAD21		yes
rs1510229	rs1018234	0.98	1.00	0.2734			yes
rs420038	rs1018234	0.98	1.00	0.2724	POLR2A, CTCF, TCF12, YY1, NR3C1, RCOR1, MAX, CREB1, REST, JUND, MYC, EP300, SMC3, FOS, JUN, FOSL2, CEBPB, STAT3, FOXA2		yes
rs394352	rs1018234	0.97	0.99	0.2734	EP300, FOSL2STAT3, FOXA2, CTCF		yes
rs112842440	rs112842440	1.00	1.00	0.0159	CTCF	OREG0017773 (CTCF)	
rs117016271	rs117162385	1.00	1.00	0.0030			yes
rs12204009	rs12204009	1.00	1.00	0.0060	STAT3		
rs12529361	rs12529361	1.00	1.00	0.2197	MYBL2, TEAD4, HNF4 in immediate vicinity (approx. 70 bases)		
rs139699952	rs139699952	1.00	1.00	0.0457	SRF		
rs9457927	rs140570886	0.81	0.90	0.0099			yes
rs7452960	rs140570886	0.81	0.90	0.0099	POLR2A, E2F1, ELF1, TAF1, MAX, E2F6, PHF8, CCNT2, REST, UBTF, CTCF, GABPA, CTCFL, EGR1, BACH1	OREG1390505 (CTCF)	yes
rs141463285	rs141463285	1.00	1.00	0.0050	CEBPB		
rs41270992	rs141550222	1.00	1.00	0.0338		OREG1390505 (CTCF)	
rs116857939	rs143665477	1.00	1.00	0.0099			yes
rs147402310	rs147402310	1.00	1.00	0.0020	ATF2		
rs149302195	rs149302195	1.00	1.00	0.0040			yes
rs150571318	rs150571318	1.00	1.00	0.0050	SETD1, CBX3		
rs9457927	rs1510224	1.00	1.00	0.0099			yes
rs7452960	rs1510224	1.00	1.00	0.0099	POLR2A, E2F1, ELF1, TAF1, MAX, E2F6, PHF8, CCNT2, REST, UBTF, CTCF, GABPA, CTCFL, EGR1, BACH1	OREG1513079 (EGR1)	yes

Proxy SNP (r^2>0.8)	Lead SNP (model 1 or 2)	R2	d'	MAF	Located in TFBS element	Located in ORegAnno element	Located in DNase hypersensitive cluster
rs60249401	rs151181359	1.00	1.00	0.0020			yes
rs115595521	rs151181359	1.00	1.00	0.0020	USF1		yes
rs145355428	rs151181359	1.00	1.00	0.0020			yes
rs116489382	rs151181359	1.00	1.00	0.0020			yes
rs148998205	rs151181359	1.00	1.00	0.0020	TFAP2C, STAT1, POLR2A, RBBP5, MYC, MAX, STAT3, TAF1, FOS, BHLHE40, GABPA	OREG1875423 (STAT1)	yes
rs149165185	rs151181359	1.00	1.00	0.0020	SETDB1		
rs112513709	rs151181359	1.00	1.00	0.0020			yes
rs6923666	rs151181359	1.00	1.00	0.0020	YY1		
rs141909377	rs151181359	1.00	1.00	0.0020	REST, EP300, FOXA1, FOXA2		
rs114561156	rs151181359	1.00	1.00	0.0020			yes
rs1510227	rs151181359	1.00	1.00	0.0020	NFIC, JUND, JUN, FOS, EP300, TCF12, TCF7L2, NR3C1, REST, ZBTB33, ETS1, GABPA, ATF3, SIX5, JUNB, FOSL2, ARID3A, BCL3, STAT3, CEBPB	OREG1627348 (FOS)	yes
rs16891557	rs151181359	1.00	1.00	0.0020	POLR2A, YY1, CTCF, RAD21		yes
rs80199309	rs151181359	1.00	1.00	0.0020	TCF12, BATF		yes
rs116059768	rs151181359	1.00	1.00	0.0020			yes
rs16891631	rs151181359	1.00	1.00	0.0020	CTCF, RAD21, TEAD4	OREG1390503 (CTCF)	yes
rs143153993	rs151181359	1.00	1.00	0.0020			yes
rs73593001	rs151181359	1.00	1.00	0.0020			yes
rs138671474	rs151181359	1.00	1.00	0.0020		OREG0017773 (CTCF)	
rs577585402	rs151181359	1.00	1.00	0.0020	NR2C2		
rs41264330	rs151181359	1.00	1.00	0.0020			yes
rs149903141	rs151181359	1.00	1.00	0.0020			yes
rs144967663	rs151181359	1.00	1.00	0.0020			yes
rs144786677	rs151181359	1.00	1.00	0.0020	CTCF		yes
rs73782428	rs151181359	1.00	1.00	0.0020	RCOR1		yes
rs4252043	rs151181359	1.00	1.00	0.0020		OREG1577498 (FOXA1)	
rs73782431	rs151181359	1.00	1.00	0.0020	MYBL2		
rs117941373	rs182532458	0.83	1.00	0.0050			yes

Proxy SNP (r^2>0.8)	Lead SNP (model 1 or 2)	R2	d'	MAF	Located in TFBS element	Located in ORegAnno element	Located in DNase hypersensitive cluster
rs189481237	rs182532458	0.83	1.00	0.0050	POLR2A, WRNIP1	OREG1265628 (SMARCA4)	
rs182532458	rs182532458	1.00	1.00	0.0060	GATA1		
rs185653030	rs182532458	1.00	1.00	0.0060			yes
rs186696265	rs186696265	1.00	1.00	0.0129			yes
rs187270959	rs187270959	1.00	1.00	0.0050			yes
rs4709485	rs2140650	0.93	0.97	0.2197		OREG1265046 (SMARCA5)	
rs1001808	rs2140650	0.95	0.99	0.2207	POLR2A		
rs4709488	rs2140650	0.94	0.98	0.2197	KAP1, GATA2, GATA3		yes
rs410926	rs2246531	0.88	0.94	0.2266			yes
rs2661836	rs2246531	0.87	0.94	0.2276			yes
rs2665354	rs2246531	0.88	0.94	0.2266			yes
rs2665353	rs2246531	0.88	0.94	0.2266			yes
rs2661846	rs2246531	0.85	0.93	0.2316			yes
rs2457557	rs2246531	0.86	0.93	0.2296			yes
rs437865	rs2246531	0.88	0.94	0.2276	FOS		yes
rs986666	rs2246531	0.97	0.99	0.2256	POLR2A		
rs2246531	rs2246531	1.00	1.00	0.2286	CEBPB	OREG1306811	
rs2136043	rs2246531	0.82	0.96	0.2097			yes
rs2504925	rs2246531	0.83	0.97	0.2068		OREG0017771 (CTCF)	
rs3004077	rs2457557	0.84	-0.96	0.2147			yes
rs2484241	rs2457557	0.87	-0.99	0.2107			yes
rs2504919	rs2457557	0.87	-0.99	0.2107	EZH2		yes
rs2504918	rs2457557	0.87	-0.99	0.2107			yes
rs410926	rs2457557	0.98	1.00	0.2266			yes
rs2661836	rs2457557	0.98	0.99	0.2276			yes
rs2665354	rs2457557	0.98	1.00	0.2266			yes
rs2665353	rs2457557	0.98	1.00	0.2266			yes
rs2661846	rs2457557	0.96	0.98	0.2316			yes
rs2457557	rs2457557	1.00	1.00	0.2296			yes

Proxy SNP (r^2>0.8)	Lead SNP (model 1 or 2)	R2	d'	MAF	Located in TFBS element	Located in ORegAnno element	Located in DNase hypersensitive cluster
rs437865	rs2457557	0.98	0.99	0.2276	FOS		yes
rs986666	rs2457557	0.89	0.95	0.2256	POLR2A		
rs2246531	rs2457557	0.86	0.93	0.2286	CEBPB	OREG1306811	
rs2076828	rs2457574	1.00	1.00	0.4344			yes
rs2504926	rs2457574	0.99	1.00	0.4354		OREG0017771 (CTCF)	
rs2457564	rs2457574	0.95	0.98	0.4334		OREG0017771 (CTCF)	
rs35358746	rs3798221	1.00	1.00	0.2117	YY1, MAFK, MAFF, ZBTB7A		
rs41259144	rs41259144	1.00	1.00	0.0119		OREG1513079 (EGR1)	
rs34126283	rs4252109	0.93	0.98	0.3002	GATA2		yes
rs4252107	rs4252109	1.00	1.00	0.2932			yes
rs4252125	rs4252109	0.99	1.00	0.2962	CTCF, TEAD4, RUNX3, RAD21	OREG1368664 (CTCF)	yes
rs4252126	rs4252109	0.99	1.00	0.2962	CTCF, TEAD4, RUNX3, RAD21		
rs4252135	rs4252109	0.98	1.00	0.2972	CTCF, ZNF143, RELA, RAD21, SMC3, FOXA1, FOXA2, ZNF263	OREG1368665 (CTCF),OREG1343765 (CTCF)	yes
rs56093624	rs4252109	0.98	1.00	0.2972	PML, MTA3, NFATC1, EBF1, CTCF, IKZF1, STAT5A, RUNX3, TCF3, ATF2, RELA, BCL11A, NFIC, TBL1XR1, BCL3, FOXM1		yes
rs4252150	rs4252109	0.93	1.00	0.3082	STAT3, MYC		
rs4252151	rs4252109	0.93	1.00	0.3082	STAT3, MYC		
rs9458022	rs4252109	0.83	0.97	0.2694	ZNF143		
rs56262039	rs4252109	0.83	0.97	0.2684	SETDB1		
rs520829	rs520829	1.00	1.00	0.4811		OREG1517229 (EGR1)	yes
rs555754	rs520829	0.96	0.99	0.4761	POLR2A, TBP, YY1, HNF4A, TAF1, EZH2	OREG1517229 (EGR1)	yes
rs668871	rs520829	0.95	0.99	0.4742	POLR2A, EZH2, ZNF263, CHD1, RBBP5, NR3C1, REST, CTCF	OREG1517229 (EGR1)	yes
rs474513	rs520829	0.93	0.97	0.4831	POLR2A, EZH2, CHD1, TCF7L2, TAF1, MAX, SIN3AK20, CEBPD, TBP, NFIC, TEAD4, HDAC2, REST, RAD21, ELF1, RXRA, FOXA2, SP1, HNF4A, CEBPB, HNF4G	OREG1517229 (EGR1),OREG1752401 (HNF4A),OREG1728897(HNF4A)	yes
rs539298	rs520829	0.93	0.97	0.4831	POLR2A, EZH2, CHD1, TCF7L2, TAF1, MAX, SIN3AK20, CEBPD, TBP, NFIC, TEAD4, HDAC2, REST, RAD21, ELF1, RXRA, FOXA2, SP1, HNF4A, CEBPB, HNF4G, EP300	OREG1517229 (EGR1),OREG1752401 (HNF4A),OREG1728897(H	yes

Proxy SNP (r^2>0.8)	Lead SNP (model 1 or 2)	R2	d'	MAF	Located in TFBS element	Located in ORegAnno element	Located in DNase hypersensitive cluster
						NF4A)	
rs655185	rs520829	0.93	0.97	0.4831	TCF7L2, NFIC, TEAD4, REST, SP1, HNF4A	OREG1752401 (HNF4A),OREG1728897(HNF4A)	
rs541091	rs520829	0.93	0.97	0.4831	TCF7L2, NFIC, TEAD4, REST, SP1, HNF4A	OREG1752401 (HNF4A),OREG1728897(HNF4A)	yes
rs506823	rs520829	0.93	0.97	0.4841			yes
rs639426	rs520829	0.93	0.97	0.4831			yes
rs509707	rs520829	0.93	0.97	0.4831			yes
rs539958	rs520829	0.93	0.97	0.4831			yes
rs624319	rs520829	0.91	0.96	0.4811			yes
rs416879	rs520829	0.93	0.97	0.4831			yes
rs695174	rs520829	0.93	0.97	0.4831			yes
rs543159	rs520829	0.90	0.96	0.4781	POLR2A, FOSL2		yes
rs675162	rs520829	0.92	0.97	0.4861	POLR2A, FOSL2, MYC, FOS, STAT3, GABPA, CEBPB, USF1	OREG1529878 (ESR1)	yes
rs9364552	rs520829	0.86	-0.96	0.4990	POLR2A		yes
rs6905958	rs520829	0.81	-0.95	0.4950	FOS		yes
rs3123634	rs520829	0.81	-0.95	0.4950	CTCF		
rs2313453	rs520829	0.81	-0.94	0.4960	POLR2A, CTCF, TCF12, YY1, NR3C1, RCOR1, MAX, CREB1, REST, JUNBD, MYC, EP300, RAD21, SMC3, FOS, JUN, FOSL2, CEBPB		yes
rs2136676	rs520829	0.81	-0.94	0.4960	TCF12, EP300, FOSL2, FOXA2, CTCF		yes
rs3105748	rs520829	0.81	-0.94	0.4960			yes
rs4709426	rs520829	0.81	-0.94	0.4970	POLR2A		
rs56393506	rs56393506	1.00	1.00	0.1819	KAP1, CTCF		
rs34126283	rs59614420	0.94	0.98	0.3002	GATA2		yes
rs4252107	rs59614420	0.99	1.00	0.2932			yes
rs4252125	rs59614420	0.97	0.99	0.2962	CTCF, TEAD4, RUNX3, RAD21	OREG1368664 (CTCF)	yes
rs4252126	rs59614420	0.97	0.99	0.2962	CTCF, TEAD4, RUNX3, RAD21		
rs4252135	rs59614420	0.97	0.99	0.2972	CTCF, ZNF143, RELA, RAD21, SMC3, FOXA1, FOXA2,	OREG1368665	yes

Proxy SNP (r^2>0.8)	Lead SNP (model 1 or 2)	R2	d'	MAF	Located in TFBS element	Located in ORegAnno element	Located in DNase hypersensitive cluster
					ZNF263	(CTCF),OREG1343765 (CTCF)	
rs56093624	rs59614420	0.97	0.99	0.2972	PML, MTA3, NFATC1, EBF1, CTCF, IKZF1, STAT5A, RUNX3, TCF3, ATF2, RELA, BCL11A, NFIC, TBL1XR1, BCL3, FOXM1		yes
rs4252150	rs59614420	0.94	1.00	0.3082	STAT3, MYC		
rs4252151	rs59614420	0.94	1.00	0.3082	STAT3, MYC		
rs9458022	rs59614420	0.82	0.97	0.2694	ZNF143		
rs56262039	rs59614420	0.82	0.97	0.2684	SETDB1		
rs614754	rs614754	1.00	1.00	0.0159			yes
rs60249401	rs6902316	1.00	1.00	0.0020			yes
rs115595521	rs6902316	1.00	1.00	0.0020	USF1		yes
rs145355428	rs6902316	1.00	1.00	0.0020			yes
rs116489382	rs6902316	1.00	1.00	0.0020			yes
rs148998205	rs6902316	1.00	1.00	0.0020	TFAP2C, STAT1, POLR2A, RBBP5, MYC, MAX, STAT3, TAF1, FOS, BHLHE40, GABPA	OREG1875423 (STAT1)	yes
rs149165185	rs6902316	1.00	1.00	0.0020	SETDB1		
rs112513709	rs6902316	1.00	1.00	0.0020			yes
rs6923666	rs6902316	1.00	1.00	0.0020	YY1		
rs141909377	rs6902316	1.00	1.00	0.0020	REST, EP300, FOXA1, FOXA2		
rs114561156	rs6902316	1.00	1.00	0.0020			yes
rs1510227	rs6902316	1.00	1.00	0.0020	NFIC, JUND, JUN, FOS, EP300, TCF12, TCF7L2, NR3C1, REST, ZBTB33, ETS1, GABPA, ATF3, SIX5, JUNB, FOSL2, ARID3A, BCL3, STAT3, CEBPB	OREG1627348 (FOS)	yes
rs16891557	rs6902316	1.00	1.00	0.0020	POLR2A, YY1, CTCF, RAD21		yes
rs80199309	rs6902316	1.00	1.00	0.0020	TCF12, BATF		yes
rs116059768	rs6902316	1.00	1.00	0.0020			yes
rs16891631	rs6902316	1.00	1.00	0.0020	CTCF, RAD21, TEAD4	OREG1390503 (CTCF)	yes
rs143153993	rs6902316	1.00	1.00	0.0020			yes
rs73593001	rs6902316	1.00	1.00	0.0020			yes
rs138671474	rs6902316	1.00	1.00	0.0020		OREG0017773 (CTCF)	
rs577585402	rs6902316	1.00	1.00	0.0020	NR2C2		

Proxy SNP (r^2>0.8)	Lead SNP (model 1 or 2)	R2	d‘	MAF	Located in TFBS element	Located in ORegAnno element	Located in DNase hypersensitive cluster
rs41264330	rs6902316	1.00	1.00	0.0020			yes
rs149903141	rs6902316	1.00	1.00	0.0020			yes
rs144967663	rs6902316	1.00	1.00	0.0020			yes
rs144786677	rs6902316	1.00	1.00	0.0020	CTCF		yes
rs73782428	rs6902316	1.00	1.00	0.0020	RCOR1		yes
rs4252043	rs6902316	1.00	1.00	0.0020		OREG1577498 (FOXA1)	
rs73782431	rs6902316	1.00	1.00	0.0020	MYBL2		
rs75234242	rs75234242	1.00	1.00	0.0348	MAFK, MAFF		
rs41272050	rs75692336	0.84	0.97	0.1491	MAFK, MAFF		
rs138874197	rs75692336	0.95	1.00	0.1282			yes
rs111657849	rs75692336	0.96	0.98	0.1332			yes
rs17557261	rs75692336	0.97	0.98	0.1342	no TFBS detected in ENCODE, but SNP is located in the cAMP response region, which mediates Lp(a) lowering effects of niacin as described by Chennamsetty et al.(40), 2012		
rs3106164	rs7769879	0.94	0.99	0.3410			yes
rs388170	rs7769879	0.94	0.99	0.3410	MAX, USF1, USF2		
rs1810126	rs7769879	1.00	1.00	0.3529	REST		
rs3106162	rs7769879	0.98	1.00	0.3569		OREG1221620(SMARCA4)	yes
rs10755578	rs9365169	0.92	1.00	0.4920			yes
rs66801308	rs9457778	0.82	0.98	0.2475	JUND		yes
rs9365116	rs9457778	0.82	0.98	0.2475	ZNF274		
rs12665079	rs9457778	0.83	0.99	0.2495	MTA3, IKZF1, NFATC1, ELA, BCL3, MAX, FOXM1, TBP, EP300, SP1, POU2F2, JUND, CHD2, MAZ, TBL1XR1, NFIC, STAT3, MXI1, ATF2, PAX5, BATF, RUNX3, IRF4, MEF2A, TCF12, EBF1, SMC3, ELK1, PSI1, BCL11A		yes
rs7767641	rs9457778	0.83	0.99	0.2495	MTA3, IKZF1, NFATC1, ELA, BCL3, MAX, FOXM1, TBP, EP300, SP1, POU2F2, JUND, CHD2, MAZ, TBL1XR1, NFIC, STAT3, MXI1, ATF2, PAX5, BATF, RUNX3, IRF4, MEF2A, TCF12, EBF1, SMC3, ELK1, PSI1, BCL11A		yes
rs9457777	rs9457778	0.83	0.99	0.2495			yes

Proxy SNP (r^2>0.8)	Lead SNP (model 1 or 2)	R2	d‘	MAF	Located in TFBS element	Located in ORegAnno element	Located in DNase hypersensitive cluster
rs4709386	rs9457778	0.84	1.00	0.2495	GATA2		yes
rs9456480	rs9457778	0.84	1.00	0.2495	JUND		yes
rs1159875	rs9457778	0.84	1.00	0.2495	CEPBP	OREG1522389 (ESR1)	yes
rs9457785	rs9457778	0.84	1.00	0.2495	SETDB1		
rs9457786	rs9457778	0.84	1.00	0.2495	SETDB1		
rs6901166	rs9457778	0.84	1.00	0.2495			yes
rs7749516	rs9457778	0.84	1.00	0.2495	MAFK		
rs1001357	rs9457778	0.84	1.00	0.2505	EBF1		yes
rs9456483	rs9457778	0.84	1.00	0.2495	MYC, MAX, USF1, MAFF, MAFK		yes
rs4252045	rs9458010	0.80	0.97	0.4831		OREG1577498 (FOXA1)	
rs2314851	rs9458010	0.81	0.98	0.4851	FOXA1		

Table S10: Results of the hypothesis-free genome-wide gene-based analysis sorted by p-value of model 1. Only significant genes are reported (significance level 1.99×10^{-6}).

Gene	Chr	Gene-based p-value based on	
		Model 1 (adjusted for age, sex)	Model 2 (adjusted for age, sex, isoforms)
LPA	6	< 1E-300	3.27E-227
PLG	6	1.53E-231	2.79E-218
LPAL2	6	1.1E-168	1.77E-175
SLC22A3	6	2.78E-151	3.53E-171
SLC22A2	6	2.36E-102	3.97E-104
MAP3K4	6	2.29E-91	2.92E-92
SLC22A1	6	2.06E-74	3.18E-69
LOC729603	6	5.62E-51	1.40E-49
IGF2R	6	3.29E-50	1.63E-48
AGPAT4	6	1.73E-48	6.13E-51
AIRN	6	3.11E-30	2.39E-29
AGPAT4-IT1	6	3.63E-22	5.49E-17
PNLDC1	6	1.24E-19	5.09E-22
MAS1	6	2.75E-09	8.51E-07
SOD2	6	1.51E-08	
PVRL2	19	1.69E-08	1.40E-07
APOC1	19	1.76E-08	2.00E-07
TOMM40	19	2.81E-08	2.67E-07
LOC100129518	6	2.83E-08	
APOE	19	2.98E-08	2.87E-07
ACAT2	6	3.15E-08	
APOC1P1	19	3.31E-08	3.52E-07
WTAP	6	3.44E-08	
PARK2	6		1.32E-08

Table S11: Results of the gene-based candidate gene analysis sorted by p-value of model 1. Results for all 21 evaluated candidate genes are reported (significance level 0.05/21=0.0024).

Gene	Chr	Gene-based p-value based on	
		Model 1 (adjusted for age, sex)	Model 2 (adjusted for age, sex, isoforms)
TLR2	4	0.00034	0.03226
PCSK9	1	0.06972	0.53516
LRP8	1	0.09064	0.93752
SREBF2	22	0.13063	0.30127
HNF1a	12	0.1405	0.35703
TLR4	9	0.15779	0.32374
FGFR4	5	0.21134	0.83208
ANXA2	15	0.56234	0.4203
LDLR	19	0.7646	0.82673
TLR6	4	0.78469	0.26427
LGALS1	22	0.80678	0.52011
CD36	7	0.86773	0.94173
IL6	7	0.86789	0.78302
FBLN5	14	0.87591	0.95648
SREBF1	17	0.91322	0.39603
LRP2	2	0.91866	0.46836
FGF19	11	0.96328	0.94088
SCARB1	12	0.96432	0.87328
NR1H4	12	0.97445	0.80344
LRP1	12	0.99222	0.94894
VLDLR	9	0.99482	0.98335

Table S12: Overview on studies that investigated an association of the apolipoprotein E polymorphism with Lp(a) concentrations.

Reference	Study type	Sample size	Finding
de Knijff et al. (1991) (41)	Cross-sectional	303 healthy persons	E2 decreased Lp(a) by 25% and E4 increased Lp(a) by 26%
Muls et al. (1993) (42)	Case study	54 normolipidemic obese women	No association
Horita et al. (1993) (43)	Cross-sectional	305 civil service workers	*APOE2* was associated with significantly lower Lp(a) in women (tendency to lower levels in men)
Schaefer et al. (1994) (44)	Population-based cohort	2258 men and women of the Framingham Offspring Study	No association
Tiret et al. (1994) (45)	Family-based study	Offsprings of 635 MI cases and 1259 controls	Carriers of *APOE2* had lower Lp(a) levels
Lindahl et al. (1994) (46)	Patients with familial hypercholesterolemia (FH)	149 patients with heterozygous FH	*APOE2* associated with lower Lp(a) levels
Heng et al. (1995) (47)	Cross-sectional	536 healthy Chinese in Singapore	No significant effect of *APOE* polymorphism
Muros et al. (1996) (48)	Cross-sectional	397 individuals of a Spanish working population of Tenerife	No association
Klausen et al. (1996) (49)	Population-based general population	466 white men with different apo(a) phenotypes	No overall effect. In apo(a) S4 isoforms Lp(a) was highest in *APOE* E3/E4. In apo(a) S2 isoforms Lp(a) was lowest in *APOE* E2/E3 genotypes.
Boomsma et al. (2000) (50)	Family Study	142 Dutch families	*APOE* did not contribute to Lp(a) variation
Frikke-Schmidt et al. (2000) (51)	Population-based general population	5,025 women and 4,035 men from the Copenhagen City Heart Study	Lower Lp(a) levels in *APOE2* carriers in women only, but not in men
Anuurad et al. (2007) (52)	Patients scheduled for diagnostic coronary arteriography	231 African Americans and 336 Caucasians	No association in Caucasians. In African Americans Lp(a) was lower in *APOE2* carriers especially in case of large apo(a) isoforms
Li et al. (2015) (53)	Genome-wide association study	2895 African Americans of the Jackson Heart Study	single nonsynonymous SNP in *APOE* significantly associated with Lp(a)
Moriarty et al. (2017) (54)	Cross-sectional	431,239 patients with laboratory testing as part of routine care	Mean Lp(a) levels were 65% higher in *APOE* E4/E4 compared with *APOE* E2/E2 and increased according to *APOE* genotype (E2/E2 < E2/E3 < E3/E3 < E3/E4 < E4/E4)

Table S13: Association results with inverse normal transformed Lp(a) levels as well as CAD risk (derived from the CARDIoGRAMplusC4D consortium) of all 40 SNPs, which were identified in model 1 (age- and sex-adjusted) and which were available in the CARDIoGRAMplusC4D results. Effect estimates and Odds Ratios refer to the minor allele. All SNPs, which are also genome-wide significantly associated with CAD risk are marked in bold. SNPs are sorted by CAD risk p-value.

		Association with inverse normal transformed Lp(a) levels			Association with CAD risk (derived from CARDIoGRAMplusC4D consortium)		
SNP	Minor/ Major allele	β	95% Confidence Interval	p-value	Odds Ratio	95% Confidence Interval	p-value
**rs118039278**	A/G	1.22	[1.16, 1.28]	1.36e-396	1.36	[1.29, 1.42]	**4.38E-37**
**rs4252185**	C/T	0.92	[0.86, 0.98]	5.31E-211	1.34	[1.28, 1.41]	**1.64E-32**
**rs186696265**	T/C	2.02	[1.90, 2.14]	4.15E-231	1.73	[1.58, 1.91]	**3.35E-30**
**rs56393506**	T/C	0.68	[0.64, 0.72]	7.76E-285	1.16	[1.13, 1.19]	**2.37E-25**
**rs7412 (*APOE2*)**	T/C	-0.19	[-0.25, -0.13]	3.47E-10	0.87	[0.84, 0.91]	**8.17E-11**
**rs7770628**	C/T	0.18	[0.16, 0.20]	4.80E-45	1.06	[1.04, 1.08]	**7.52E-09**
**rs1510224**	C/T	1.35	[1.25, 1.45]	4.27E-160	1.40	[1.25, 1.57]	**8.22E-09**
rs4252198	G/C	1.15	[1.03, 1.27]	2.72E-84	1.27	[1.16, 1.39]	3.58E-07
rs9458010	C/T	0.09	[0.06, 0.12]	4.11E-12	1.05	[1.03, 1.08]	2.30E-06
rs41269133	C/T	-0.33	[-0.37, -0.29]	5.42E-55	0.93	[0.90, 0.96]	4.29E-06
rs184278183	T/C	0.95	[0.80, 1.10]	9.36E-34	1.28	[1.15, 1.43]	9.71E-06
rs520829	G/T	-0.14	[-0.16, -0.12]	2.65E-29	0.96	[0.95, 0.98]	5.82E-05
rs2457574	A/G	-0.21	[-0.23, -0.19]	2.77E-63	0.96	[0.95, 0.98]	7.54E-05
rs78439586	A/G	-0.42	[-0.48, -0.36]	1.28E-47	0.91	[0.86, 0.95]	7.73E-05
rs73596816	A/G	0.66	[0.51, 0.81]	1.53E-18	1.14	[1.06, 1.21]	0.0002
rs1018234	T/C	-0.12	[-0.15, -0.09]	6.30E-16	0.97	[0.95, 0.99]	0.0005
rs141550222	G/C	-0.54	[-0.62, -0.46]	6.73E-44	0.89	[0.84, 0.95]	0.0009
rs4252109	G/T	-0.29	[-0.32, -0.26]	4.86E-105	0.97	[0.95, 0.99]	0.0023
rs6902316	G/A	0.74	[0.61, 0.87]	4.30E-29	1.13	[1.04, 1.22]	0.0041
rs12529361	C/T	0.30	[0.27, 0.33]	5.57E-69	1.04	[1.01, 1.07]	0.0051
rs6938647	A/C	-0.37	[-0.4, -0.34]	7.48E-119	0.96	[0.94, 0.99]	0.0079
rs62440901	T/C	0.14	[0.10, 0.18]	3.11E-14	1.03	[1.01, 1.06]	0.0093
rs41272114	T/C	-0.78	[-0.86, -0.7]	4.74E-86	0.93	[0.88, 0.99]	0.0204
rs147402310	G/T	-0.63	[-0.74, -0.52]	8.97E-32	0.91	[0.84, 0.99]	0.0225
rs614754	C/G	0.72	[0.54, 0.90]	1.43E-15	1.15	[1.02, 1.29]	0.0243
rs185934663	G/T	-0.81	[-0.99, -0.63]	4.58E-18	0.86	[0.74, 1.00]	0.0484
rs2489940	T/G	-0.75	[-0.87, -0.63]	1.67E-32	0.91	[0.83, 1.01]	0.0709
rs142126734	A/G	0.44	[0.38, 0.50]	8.52E-48	1.05	[1.00, 1.10]	0.0762
rs76000021	C/T	-0.41	[-0.52, -0.30]	4.70E-13	0.93	[0.85, 1.01]	0.0901
rs6903649	C/T	-0.20	[-0.23, -0.17]	3.91E-45	0.98	[0.96, 1.00]	0.1055
rs62441903	G/A	-0.82	[-0.96, -0.68]	4.86E-33	0.93	[0.85, 1.02]	0.1433
rs2140650	G/A	0.05	[0.02, 0.08]	1.10E-03	1.01	[0.99, 1.03]	0.2658
rs41259144	T/C	-0.85	[-1.01, -0.69]	4.45E-24	0.93	[0.79, 1.09]	0.3877
rs79246098	C/T	0.28	[0.14, 0.42]	7.53E-05	1.04	[0.93, 1.16]	0.4800
rs139699952	C/G	0.37	[0.26, 0.48]	4.70E-11	1.02	[0.95, 1.11]	0.5414
rs2457557	T/C	-0.05	[-0.08, -0.02]	5.60E-04	0.99	[0.97, 1.02]	0.5628
rs143520616	T/G	0.27	[0.17, 0.37]	5.01E-08	0.99	[0.93, 1.06]	0.7435
rs2246531	G/A	0.00	[-0.03, 0.03]	8.48E-01	1.00	[0.98, 1.03]	0.9167
rs12664092	C/A	0.20	[0.13, 0.27]	1.92E-07	1.00	[0.95, 1.06]	0.9358
rs9457778	T/C	-0.05	[-0.08, -0.02]	8.75E-04	1.00	[0.98, 1.02]	0.9413

Supplementary Figures

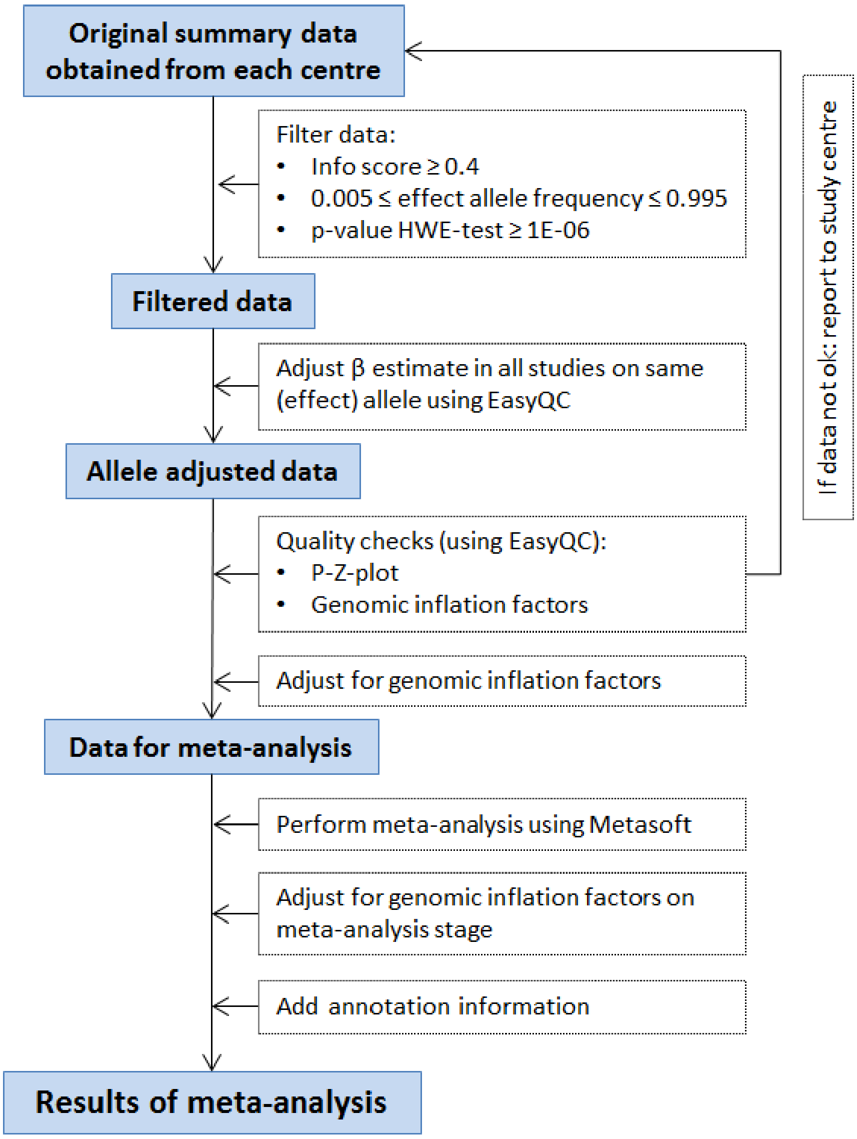

Figure S1: Quality control and meta-analysis workflow.

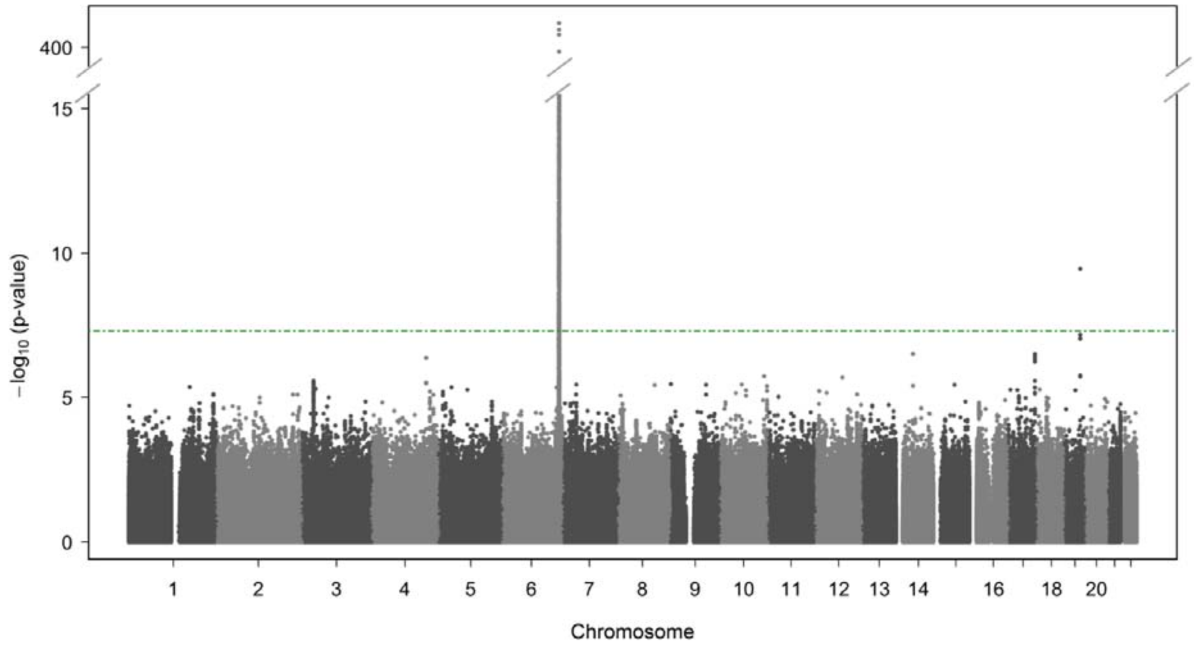

Figure S2: Manhattan-plot for the meta-analysis on inverse-normal transformed Lp(a) values, adjusted for age and sex. Results are based on five cohorts including 13,781 individuals. The y-axis is broken to display all relevant results.

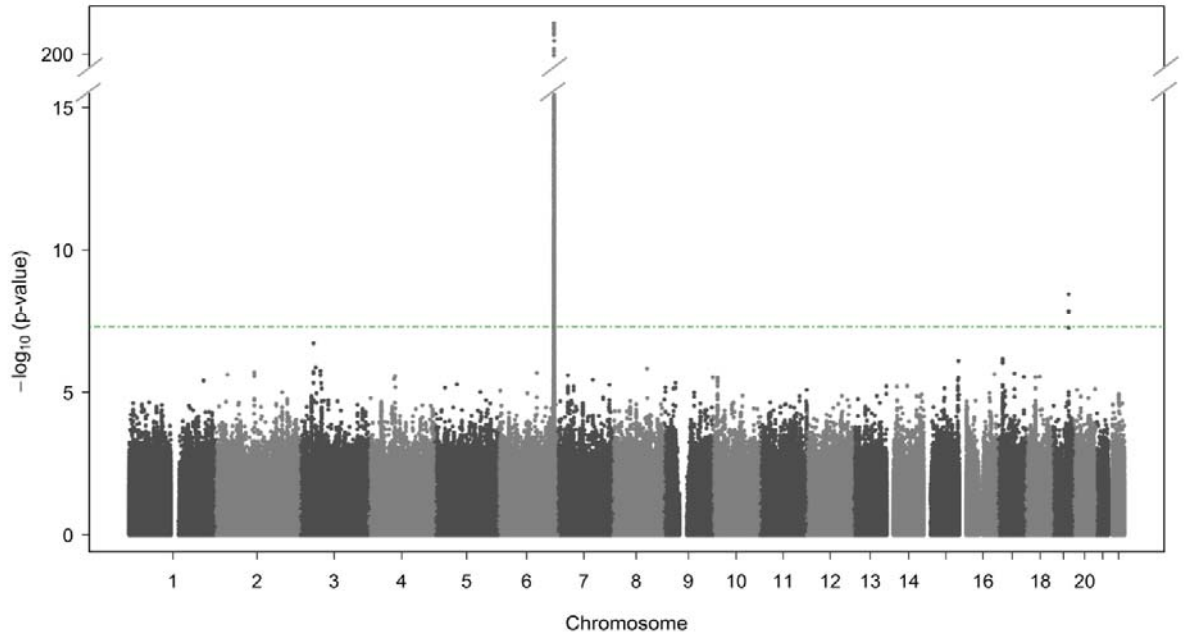

Figure S3: Manhattan-plot for the meta-analysis on inverse-normal transformed Lp(a) values, adjusted for age, sex and the predominantly expressed apo(a) isoform. Results are based on five cohorts including 13,781 individuals. The y-axis is broken to display all relevant results.

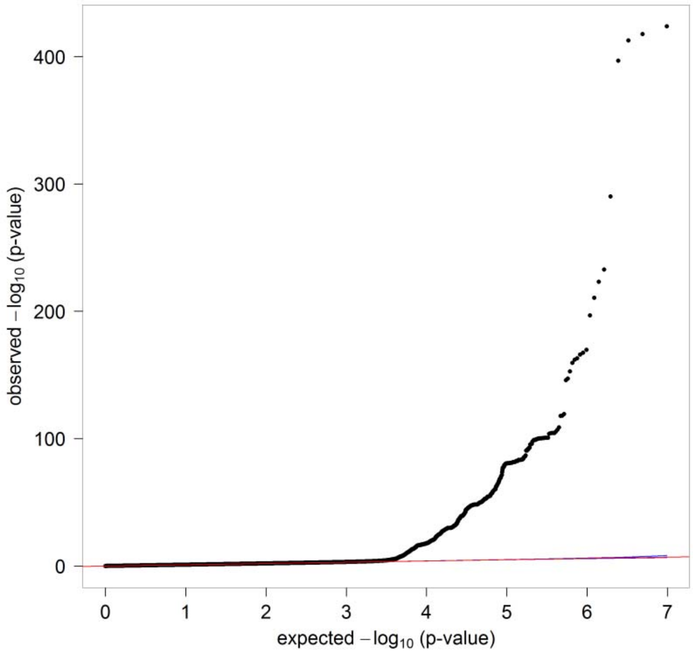

Figure S4: QQ-plot for the meta-analysis on inverse-normal transformed Lp(a) values adjusted for age and sex.

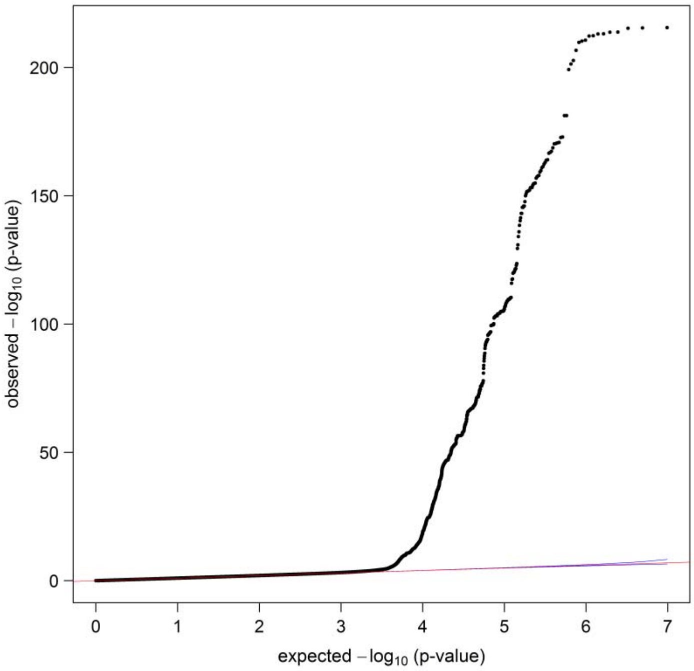

Figure S5: QQ-plot for the meta-analysis on inverse-normal transformed Lp(a) values adjusted for age, sex and the predominantly expressed apo(a) isoform.

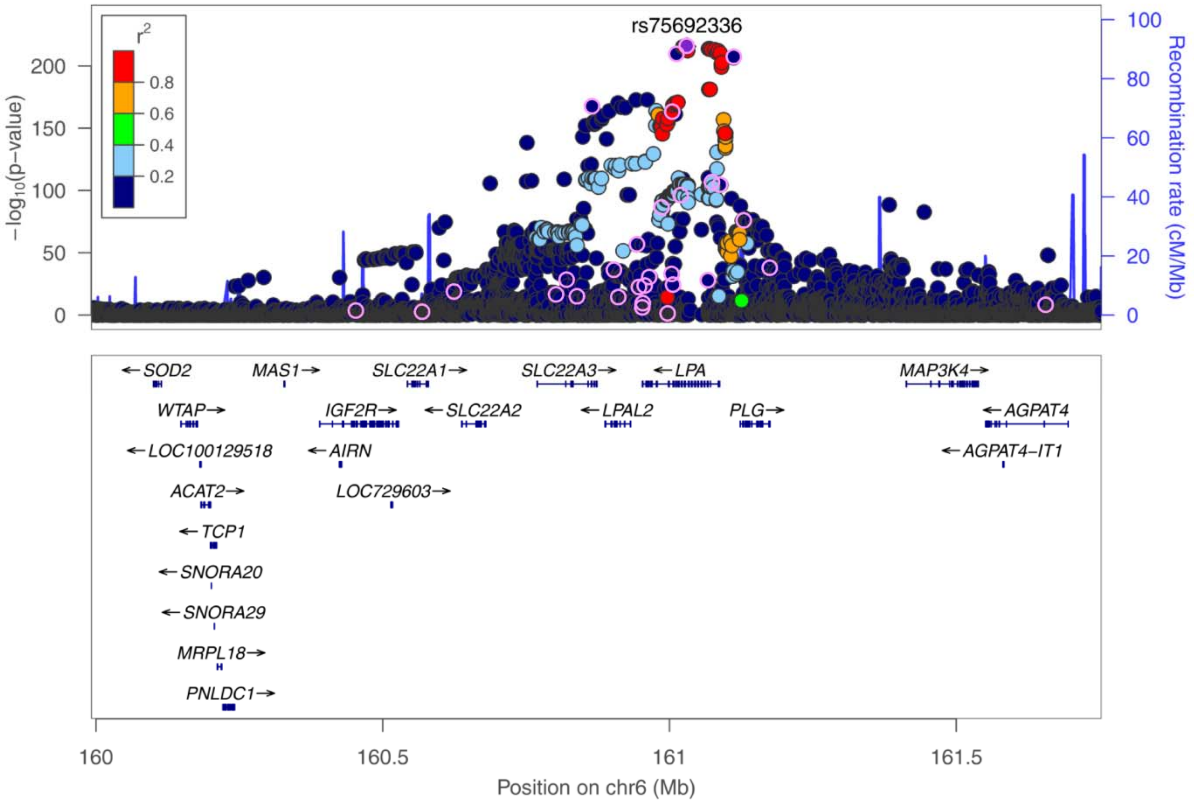

Figure S6: Regional plot showing the genomic region surrounding the *LPA* gene (chr6:159,991,850-161,753,083; LD refers to rs75692336, based on 1000G EUR); p-values are derived from the meta-analysis on the five cohorts on inverse-normal-transformed Lp(a) concentrations, adjusted for age, sex and the predominantly expressed apo(a) isoform. All SNPs, which are independently associated with Lp(a) in a joint model are circled.

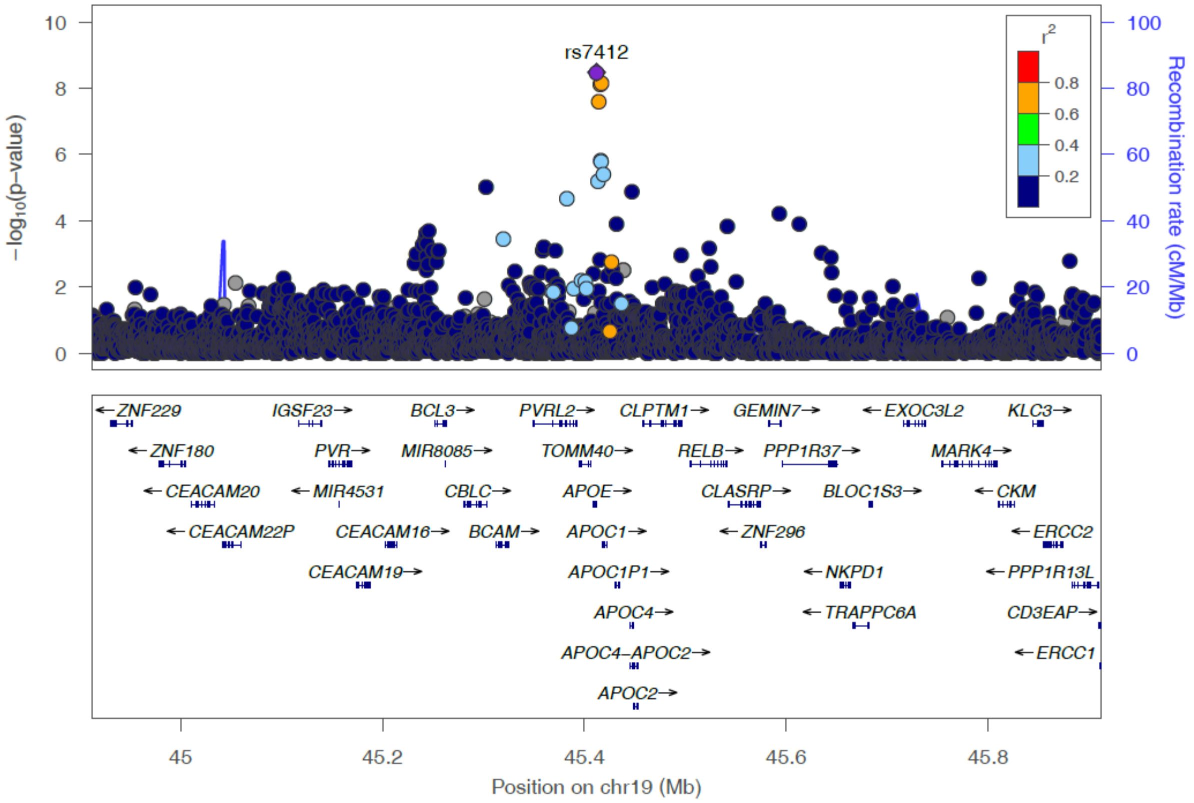

Figure S7: Regional plot showing the genomic region defined by the *APOE*-lead SNP rs7412 +/- 500 kB (LD refers to rs7412, based on 1000G EUR); p-values are derived from the meta-analysis on the five cohorts on inverse-normal-transformed Lp(a) concentrations, adjusted for age, sex and the predominantly expressed apo(a) isoform.

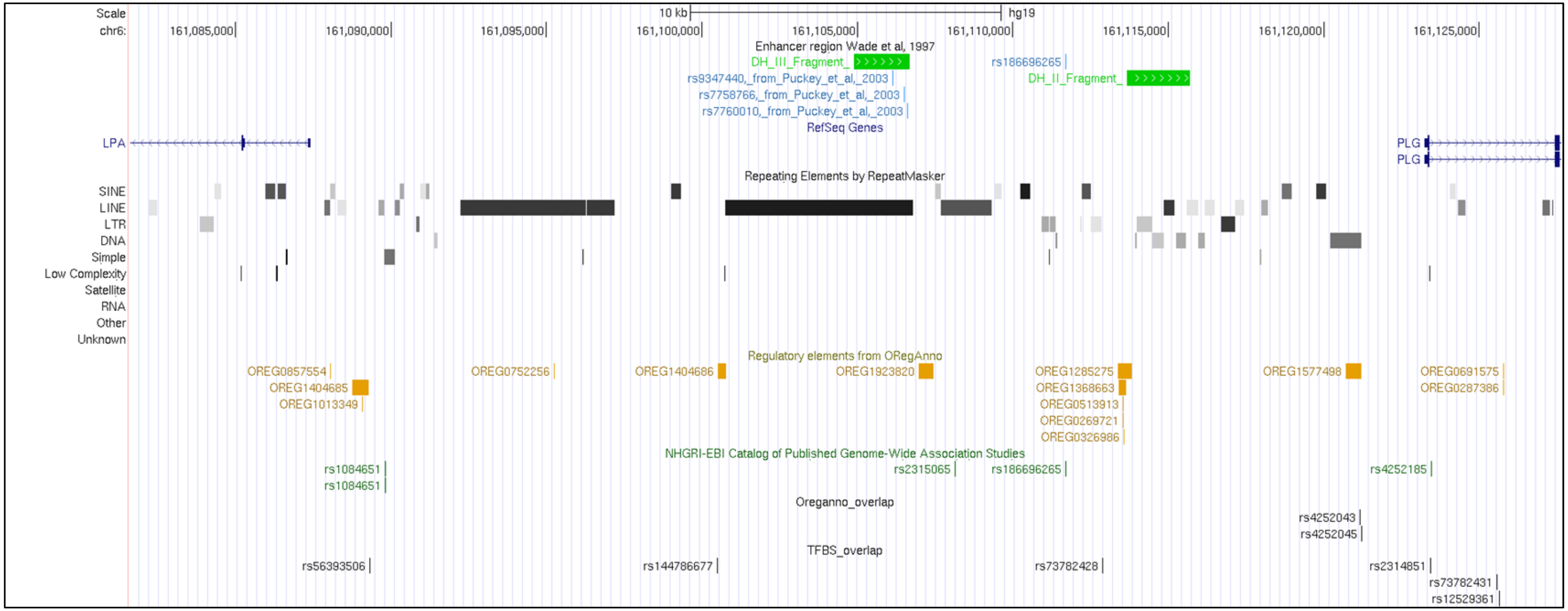

Figure S8: Intergenic region LPA-PLG with enhancer region described by Wade et al, 1997 (55).

This figure shows the intergenic region between *LPA* and *PLG*, superimposed to the UCSC Genome output for this region as described in Lamina et al, 2011 (56). The positions of the enhancer regions reported by Wade et al, 1997 is shown by green bars in the top row. SNPs are shown in blue. The second row shows the gene location, followed by repeat regions as predicted by Repeat Masker. The DHIII enhancer overlaps a LINE retrotransposon element, as determined by Wade et al, 1997 (55). The next rows show ORegAnno elements, GWAS hits in this region and the IDs of SNPs directly overlapping ORegAnno and transcription factor binding sites (TFBS) as reported Tables S4 and S9.

The three SNPs in the enhancer region reported by Puckey et al, 2003 (57) were associated with 2.5-fold increased reporter gene transcriptional activity, 0.6-fold reporter gene transcriptional activity, and 0.7-fold reporter gene transcriptional activity for rs9347440, rs7760010 and rs7758766, respectively. rs7760010 and rs7758766 were also present in our dataset (rs7760010: $p=1.38x10^{-4}$, ($p=1.38x10^{-4}$, β(sd)=0.47(0.122), rs7758766: p=6.37E-49, β(sd)=0.26(0.026)).

The figure also shows the location of the SNP rs186696265, that showed the strongest effect on Lp(a) in our study (see Supplementary Tables 5 and 6 and the Discussion section in the main document), between the two reported enhancers.

Supplementary References

1. Firmann, M., V. Mayor, P. M. Vidal, M. Bochud, A. Pécoud, D. Hayoz, F. Paccaud, M. Preisig, K. S. Song, X. Yuan, T. M. Danoff, H. A. Stirnadel, D. Waterworth, V. Mooser, G. Waeber, and P. Vollenweider. 2008. The CoLaus study: a population-based study to investigate the epidemiology and genetic determinants of cardiovascular risk factors and metabolic syndrome. *BMC Cardiovasc. Disord.* **8**: 6.

2. Higgins, M., M. Province, G. Heiss, J. Eckfeldt, R. C. Ellison, A. R. Folsom, D. C. Rao, J. M. Sprafka, and R. Williams. 1996. NHLBI Family Heart Study: objectives and design. *Am. J. Epidemiol.* **143**: 1219–28.

3. Heid, I. M., E. Boes, M. Müller, B. Kollerits, C. Lamina, S. Coassin, C. Gieger, A. Döring, N. Klopp, R. Frikke-Schmidt, A. Tybjaerg-Hansen, A. Brandstätter, A. Luchner, T. Meitinger, H.-E. Wichmann, and F. Kronenberg. 2008. Genome-wide association analysis of high-density lipoprotein cholesterol in the population-based KORA study sheds new light on intergenic regions. *Circ. Cardiovasc. Genet.* **1**: 10–20.

4. Juonala, M., J. S. A. Viikari, and O. T. Raitakari. 2013. Main findings from the prospective Cardiovascular Risk in Young Finns Study. *Curr. Opin. Lipidol.* **24**: 57–64.

5. Jylhävä, J., L.-P. Lyytikäinen, M. Kähönen, N. Hutri-Kähönen, J. Kettunen, J. Viikari, O. T. Raitakari, T. Lehtimäki, and M. Hurme. 2012. A genome-wide association study identifies UGT1A1 as a regulator of serum cell-free DNA in young adults: The Cardiovascular Risk in Young Finns Study. *PLoS One*. **7**: e35426.

6. Heid, I. M., S. A. Wagner, H. Gohlke, B. Iglseder, J. C. Mueller, P. Cip, G. Ladurner, R. Reiter, A. Stadlmayr, V. Mackevics, T. Illig, F. Kronenberg, and B. Paulweber. 2006. Genetic architecture of the APM1 gene and its influence on adiponectin plasma levels and parameters of the metabolic syndrome in 1,727 healthy Caucasians. *Diabetes*. **55**: 375–84.

7. Kronenberg, F., E. M. Lobentanz, P. König, G. Utermann, and H. Dieplinger. 1994. Effect of sample storage on the measurement of lipoprotein[a], apolipoproteins B and A-IV, total and high density lipoprotein cholesterol and triglycerides. *J. Lipid Res.* **35**: 1318–28.

8. Kronenberg, F., E. Kuen, E. Ritz, R. Junker, P. König, G. Kraatz, K. Lhotta, J. F. Mann, G. A. Müller, U. Neyer, W. Riegel, P. Reigler, V. Schwenger, and A. Von Eckardstein. 2000. Lipoprotein(a) serum concentrations and apolipoprotein(a) phenotypes in mild and moderate renal failure. *J. Am. Soc. Nephrol.* **11**: 105–15.

9. Winkler, T. W., F. R. Day, D. C. Croteau-Chonka, A. R. Wood, A. E. Locke, R. Mägi, T. Ferreira, T. Fall, M. Graff, A. E. Justice, J. Luan, S. Gustafsson, J. C. Randall, S. Vedantam, T. Workalemahu, T. O. Kilpeläinen, A. Scherag, T. Esko, Z. Kutalik, I. M. Heid, R. J. F. Loos, and Genetic Investigation of Anthropometric Traits (GIANT) Consortium. 2014. Quality control and conduct of genome-wide association meta-analyses. *Nat. Protoc.* **9**: 1192–212.

10. Han, B., and E. Eskin. 2011. Random-effects model aimed at discovering associations in meta-analysis of genome-wide association studies. *Am. J. Hum. Genet.* **88**: 586–98.

11. Romagnuolo, R., C. A. Scipione, M. B. Boffa, S. M. Marcovina, N. G. Seidah, and M. L. Koschinsky. 2015. Lipoprotein(a) catabolism is regulated by proprotein convertase subtilisin/kexin type 9 through the low density lipoprotein receptor. *J. Biol. Chem.* **290**: 11649–62.

12. Scipione, C. A., S. E. Sayegh, R. Romagnuolo, S. Tsimikas, S. M. Marcovina, M. B. Boffa, and M. L. Koschinsky. 2015. Mechanistic insights into Lp(a)-induced IL-8 expression: a role for oxidized

phospholipid modification of apo(a). *J. Lipid Res.* **56**: 2273–85.

13. Tsimikas, S., and J. L. Hall. 2012. Lipoprotein(a) as a potential causal genetic risk factor of cardiovascular disease: a rationale for increased efforts to understand its pathophysiology and develop targeted therapies. *J. Am. Coll. Cardiol.* **60**: 716–21.

14. Kalaivani, V., and P. S. Appukuttan. 2014. Circulating Lp(a):LDL complexes contain LDL molecules proportionate to Lp(a) size and bind to galectin-1: a possible route for LDL entry into cells. *Lipids*. **49**: 1101–13.

15. März, W., A. Beckmann, H. Scharnagl, R. Siekmeier, U. Mondorf, I. Held, W. Schneider, K. T. Preissner, L. K. Curtiss, and W. Gross. 1993. Heterogeneous lipoprotein (a) size isoforms differ by their interaction with the low density lipoprotein receptor and the low density lipoprotein receptor-related protein/alpha 2-macroglobulin receptor. *FEBS Lett.* **325**: 271–5.

16. Niemeier, A., T. Willnow, H. Dieplinger, C. Jacobsen, N. Meyer, J. Hilpert, and U. Beisiegel. 1999. Identification of megalin/gp330 as a receptor for lipoprotein(a) in vitro. *Arterioscler. Thromb. Vasc. Biol.* **19**: 552–61.

17. Arnold, M., J. Raffler, A. Pfeufer, K. Suhre, and G. Kastenmüller. 2015. SNiPA: an interactive, genetic variant-centered annotation browser. *Bioinformatics*. **31**: 1334–6.

18. Yang, X.-P., M. J. Amar, B. Vaisman, A. V Bocharov, T. G. Vishnyakova, L. a Freeman, R. J. Kurlander, A. P. Patterson, L. C. Becker, and A. T. Remaley. 2013. Scavenger receptor-BI is a receptor for lipoprotein(a). *J. Lipid Res.* **54**: 2450–7.

19. Argraves, K. M., K. F. Kozarsky, J. T. Fallon, P. C. Harpel, and D. K. Strickland. 1997. The atherogenic lipoprotein Lp(a) is internalized and degraded in a process mediated by the VLDL receptor. *J. Clin. Invest.* **100**: 2170–81.

20. Kapetanopoulos, A., F. Fresser, G. Millonig, Y. Shaul, G. Baier, and G. Utermann. 2002. Direct interaction of the extracellular matrix protein DANCE with apolipoprotein(a) mediated by the kringle IV-type 2 domain. *Mol. Genet. Genomics*. **267**: 440–6.

21. Berthold, H. K., M. Laudes, W. Krone, and I. Gouni-Berthold. 2011. Association between the interleukin-6 promoter polymorphism -174G/C and serum lipoprotein(a) concentrations in humans. *PLoS One*. **6**: e24719.

22. Chennamsetty, I., T. Claudel, K. M. Kostner, A. Baghdasaryan, D. Kratky, S. Levak-Frank, S. Frank, F. J. Gonzalez, M. Trauner, and G. M. Kostner. 2011. Farnesoid X receptor represses hepatic human APOA gene expression. *J. Clin. Invest.* **121**: 3724–34.

23. Chennamsetty, I., T. Claudel, K. M. Kostner, M. Trauner, and G. M. Kostner. 2012. FGF19 signaling cascade suppresses APOA gene expression. *Arterioscler. Thromb. Vasc. Biol.* **32**: 1220–7.

24. Seimon, T. A., M. J. Nadolski, X. Liao, J. Magallon, M. Nguyen, N. T. Feric, M. L. Koschinsky, R. Harkewicz, J. L. Witztum, S. Tsimikas, D. Golenbock, K. J. Moore, and I. Tabas. 2010. Atherogenic lipids and lipoproteins trigger CD36-TLR2-dependent apoptosis in macrophages undergoing endoplasmic reticulum stress. *Cell Metab.* **12**: 467–82.

25. Hoover-Plow, J., and M. Huang. 2013. Lipoprotein(a) metabolism: potential sites for therapeutic targets. *Metabolism.* **62**: 479–91.

26. Toth, P. P. 2016. PCSK9 and Lipoprotein(a): The Plot Thickens. *Circ. Res.* **119**: 3–6.

27. Seidah, N. G., Z. Awan, M. Chrétien, and M. Mbikay. 2014. PCSK9: a key modulator of cardiovascular health. *Circ. Res.* **114**: 1022–36.

28. Karolchik, D., A. S. Hinrichs, T. S. Furey, K. M. Roskin, C. W. Sugnet, D. Haussler, and W. J. Kent. 2004. The UCSC Table Browser data retrieval tool. *Nucleic Acids Res.* **32**: D493-6.

29. Yates, A., W. Akanni, M. R. Amode, D. Barrell, K. Billis, D. Carvalho-Silva, C. Cummins, P. Clapham, S. Fitzgerald, L. Gil, C. G. Girón, L. Gordon, T. Hourlier, S. E. Hunt, S. H. Janacek, N. Johnson, T. Juettemann, S. Keenan, I. Lavidas, F. J. Martin, T. Maurel, W. McLaren, D. N. Murphy, R. Nag, M. Nuhn, A. Parker, M. Patricio, M. Pignatelli, M. Rahtz, H. S. Riat, D. Sheppard, K. Taylor, A. Thormann, A. Vullo, S. P. Wilder, A. Zadissa, E. Birney, J. Harrow, M. Muffato, E. Perry, M. Ruffier, G. Spudich, S. J. Trevanion, F. Cunningham, B. L. Aken, D. R. Zerbino, and P. Flicek. 2016. Ensembl 2016. *Nucleic Acids Res.* **44**: D710-6.

30. Birney, E., J. A. Stamatoyannopoulos, A. Dutta, R. G. oacute, T. R. Gingeras, E. H. Margulies, Z. Weng, M. Snyder, E. T. Dermitzakis, R. E. Thurman, M. S. Kuehn, C. M. Taylor, S. Neph, C. M. Koch, S. Asthana, A. Malhotra, I. Adzhubei, J. A. Greenbaum, R. M. Andrews, P. Flicek, P. J. Boyle, H. Cao, N. P. Carter, G. K. Clelland, S. Davis, N. Day, P. Dhami, S. C. Dillon, M. O. Dorschner, H. Fiegler, P. G. Giresi, J. Goldy, M. Hawrylycz, A. Haydock, R. Humbert, K. D. James, B. E. Johnson, E. M. Johnson, T. T. Frum, E. R. Rosenzweig, N. Karnani, K. Lee, G. C. Lefebvre, P. A. Navas, F. Neri, S. C. J. Parker, P. J. Sabo, R. Sandstrom, A. Shafer, D. Vetrie, et al. 2007. Identification and analysis of functional elements in 1% of the human genome by the ENCODE pilot project. *Nature*. **447**: 799–816.

31. Lesurf, R., K. C. Cotto, G. Wang, M. Griffith, K. Kasaian, S. J. M. Jones, S. B. Montgomery, O. L. Griffith, and Open Regulatory Annotation Consortium. 2016. ORegAnno 3.0: a community-driven resource for curated regulatory annotation. *Nucleic Acids Res.* **44**: D126-32.

32. GTEx Consortium. 2015. Human genomics. The Genotype-Tissue Expression (GTEx) pilot analysis: multitissue gene regulation in humans. *Science*. **348**: 648–60.

33. Zannis, V. I., J. L. Breslow, G. Utermann, R. W. Mahley, K. H. Weisgraber, R. J. Havel, J. L. Goldstein, M. S. Brown, G. Schonfeld, W. R. Hazzard, and C. Blum. 1982. Proposed nomenclature of apoE isoproteins, apoE genotypes, and phenotypes. *J. Lipid Res.* **23**: 911–4.

34. Utermann, G., U. Langenbeck, U. Beisiegel, and W. Weber. 1980. Genetics of the apolipoprotein E system in man. *Am. J. Hum. Genet.* **32**: 339–47.

35. Gerstein, M. B., A. Kundaje, M. Hariharan, S. G. Landt, K.-K. Yan, C. Cheng, X. J. Mu, E. Khurana, J. Rozowsky, R. Alexander, R. Min, P. Alves, A. Abyzov, N. Addleman, N. Bhardwaj, A. P. Boyle, P. Cayting, A. Charos, D. Z. Chen, Y. Cheng, D. Clarke, C. Eastman, G. Euskirchen, S. Frietze, Y. Fu, J. Gertz, F. Grubert, A. Harmanci, P. Jain, M. Kasowski, P. Lacroute, J. Leng, J. Lian, H. Monahan, H. O'Geen, Z. Ouyang, E. C. Partridge, D. Patacsil, F. Pauli, D. Raha, L. Ramirez, T. E. Reddy, B. Reed, M. Shi, T. Slifer, J. Wang, L. Wu, X. Yang, K. Y. Yip, G. Zilberman-Schapira, et al. 2012. Architecture of the human regulatory network derived from ENCODE data. *Nature*. **489**: 91–100.

36. Wang, J., J. Zhuang, S. Iyer, X. Lin, T. W. Whitfield, M. C. Greven, B. G. Pierce, X. Dong, A. Kundaje, Y. Cheng, O. J. Rando, E. Birney, R. M. Myers, W. S. Noble, M. Snyder, and Z. Weng. 2012. Sequence features and chromatin structure around the genomic regions bound by 119 human transcription factors. *Genome Res.* **22**: 1798–812.

37. Wang, J., J. Zhuang, S. Iyer, X.-Y. Lin, M. C. Greven, B.-H. Kim, J. Moore, B. G. Pierce, X. Dong, D. Virgil, E. Birney, J.-H. Hung, and Z. Weng. 2013. Factorbook.org: a Wiki-based database for

transcription factor-binding data generated by the ENCODE consortium. *Nucleic Acids Res.* **41**: D171-6.

38. Ogorelkova, M., H. G. Kraft, C. Ehnholm, and G. Utermann. 2001. Single nucleotide polymorphisms in exons of the apo(a) kringles IV types 6 to 10 domain affect Lp(a) plasma concentrations and have different patterns in Africans and Caucasians. *Hum. Mol. Genet.* **10**: 815–24.

39. Prins, J., F. R. Leus, B. N. Bouma, and H. J. van Rijn. 1999. The identification of polymorphisms in the coding region of the apolipoprotein (a) gene--association with earlier identified polymorphic sites and influence on the lipoprotein (a) concentration. *Thromb. Haemost.* **82**: 1709–17.

40. Chennamsetty, I., K. M. Kostner, T. Claudel, M. Vinod, S. Frank, T. S. Weiss, M. Trauner, and G. M. Kostner. 2012. Nicotinic acid inhibits hepatic APOA gene expression: studies in humans and in transgenic mice. *J. Lipid Res.* **53**: 2405–12.

41. de Knijff, P., A. Kaptein, D. Boomsma, H. M. Princen, R. R. Frants, and L. M. Havekes. 1991. Apolipoprotein E polymorphism affects plasma levels of lipoprotein(a). *Atherosclerosis*. **90**: 169–74.

42. Muls, E., K. Kempen, G. Vansant, C. Cobbaert, and W. Saris. 1993. The effects of weight loss and apolipoprotein E polymorphism on serum lipids, apolipoproteins A-I and B, and lipoprotein(a). *Int. J. Obes. Relat. Metab. Disord.* **17**: 711–6.

43. Horita, K., M. Eto, M. Saito, H. Nakata, Y. Iwashima, H. Ito, M. Takahashi, A. Kondo, A. Morikawa, and I. Makino. 1993. Effects of apolipoprotein E polymorphism on plasma lipoprotein(a) levels. *Artery*. **20**: 324–36.

44. Schaefer, E. J., S. Lamon-Fava, S. Johnson, J. M. Ordovas, M. M. Schaefer, W. P. Castelli, and P. W. Wilson. 1994. Effects of gender and menopausal status on the association of apolipoprotein E phenotype with plasma lipoprotein levels. Results from the Framingham Offspring Study. *Arterioscler. Thromb. a J. Vasc. Biol.* **14**: 1105–13.

45. Tiret, L., P. de Knijff, H. J. Menzel, C. Ehnholm, V. Nicaud, and L. M. Havekes. 1994. ApoE polymorphism and predisposition to coronary heart disease in youths of different European populations. The EARS Study. European Atherosclerosis Research Study. *Arterioscler. Thromb. a J. Vasc. Biol.* **14**: 1617–24.

46. Lindahl, G., F. Mailly, S. Humphries, and M. Seed. 1994. Apolipoprotein E phenotype and lipoprotein(a) in familial hypercholesterolaemia: implication for lipoprotein(a) metabolism. *Clin. Investig.* **72**: 631–8.

47. Heng, C. K., N. Saha, and J. S. Tay. 1995. Lack of association of apolipoprotein E polymorphism with plasma Lp(a) levels in the Chinese. *Clin. Genet.* **48**: 113–9.

48. Muros, M., and C. Rodríguez-Ferrer. 1996. Apolipoprotein E polymorphism influence on lipids, apolipoproteins and Lp(a) in a Spanish population underexpressing apo E4. *Atherosclerosis*. **121**: 13–21.

49. Klausen, I. C., L. U. Gerdes, P. S. Hansen, L. Lemming, C. Gerdes, and O. Faergeman. 1996. Effects of apoE gene polymorphism on Lp(a) concentrations depend on the size of apo(a): a study of 466 white men. *J. Mol. Med. (Berl).* **74**: 685–90.

50. Boomsma, D. I., P. Knijff, A. Kaptein, C. Labeur, N. G. Martin, L. M. Havekes, and H. M. Princen. 2000. The effect of apolipoprotein(a)-, apolipoprotein E-, and apolipoprotein A4-

polymorphisms on quantitative lipoprotein(a) concentrations. *Twin Res.* **3**: 152–8.

51. Frikke-Schmidt, R., B. G. Nordestgaard, B. Agerholm-Larsen, P. Schnohr, and A. Tybjaerg-Hansen. 2000. Context-dependent and invariant associations between lipids, lipoproteins, and apolipoproteins and apolipoprotein E genotype. *J. Lipid Res.* **41**: 1812–22.

52. Anuurad, E., G. Lu, J. Rubin, T. A. Pearson, and L. Berglund. 2007. ApoE genotype affects allele-specific apo[a] levels for large apo[a] sizes in African Americans: the Harlem-Basset Study. *J. Lipid Res.* **48**: 693–8.

53. Li, J., L. A. Lange, J. Sabourin, Q. Duan, W. Valdar, M. S. Willis, Y. Li, J. G. Wilson, and E. M. Lange. 2015. Genome- and exome-wide association study of serum lipoprotein (a) in the Jackson Heart Study. *J. Hum. Genet.* **60**: 755–61.

54. Moriarty, P. M., S. A. Varvel, P. L. S. M. Gordts, J. P. McConnell, and S. Tsimikas. 2017. Lipoprotein(a) Mass Levels Increase Significantly According to APOE Genotype: An Analysis of 431 239 Patients. *Arterioscler. Thromb. Vasc. Biol.* **37**: 580–588.

55. Wade, D. P., L. H. Puckey, B. L. Knight, F. Acquati, A. Mihalich, and R. Taramelli. 1997. Characterization of multiple enhancer regions upstream of the apolipoprotein(a) gene. *J. Biol. Chem.* **272**: 30387–99.

56. Lamina, C., S. Coassin, T. Illig, and F. Kronenberg. 2011. Look beyond one's own nose: combination of information from publicly available sources reveals an association of GATA4 polymorphisms with plasma triglycerides. *Atherosclerosis*. **219**: 698–703.

57. Puckey, L. H., and B. L. Knight. 2003. Sequence and functional changes in a putative enhancer region upstream of the apolipoprotein(a) gene. *Atherosclerosis*. **166**: 119–27.
